# A Novel Approach for Dry Cutting Inconel 718 in a More Sustainable and Low-Cost Way by Actively and Purposely Utilizing the Built-Up Layer

**DOI:** 10.3390/mi14091787

**Published:** 2023-09-19

**Authors:** Xiaoqi Song, Weiming He, Tohru Ihara

**Affiliations:** 1 Department of Mechanical Systems Engineering, School of Engineering, Kogakuin University, Tokyo 192-0015, Japan; 2 School of Mechanical Engineering, University of Shanghai for Science and Technology, Shanghai 200093, China; 3 Department of Precision Mechanics, Chuo University, Tokyo 112-8551, Japan

**Keywords:** dry machining, built-up layer, self-protective effect, tool wear, Inconel 718

## Abstract

Due to its physical and mechanical properties, Inconel 718 remains a difficult-to-cut material and there is an urgent need to develop a more sustainable and low-cost way to machine it. A novel approach for dry cutting Inconel 718 by actively and purposely utilizing the built-up layer (BUL), which can be called the self-protective tool (SPT) method, is proposed and investigated in detail in this paper. Various cutting experiments were carried out using the age-treated Inconel 718 and uncoated cemented carbide tools. The formation condition of the BUL, its formation mechanism, its stability, and its protective effect were examined by measuring the tools after cutting using a scanning electron microscope (SEM) and laser confocal microscopy (LCM). The influences of BUL on the cutting process were investigated using cutting force analysis and surface roughness analysis. The results confirmed that the stability of the BUL is very high, and the BUL can not only significantly protect the tool from wear but also reduce friction at the tool–chip interface and maintain surface roughness. It also revealed that the height of the BUL can play a very important role in its protective effect. Comparative experiments verified the effectiveness and generalizability of the proposed SPT method.

## 1. Introduction

Inconel 718 has been the most commonly used and researched nickel-based superalloy in the world since its discovery in the 1960s [1,2], due to its outstanding mechanical properties, corrosion resistance, fatigue resistance, and excellent weldability. It was reported that over 50% of the weight of aircraft engines is formed by Inconel 718 [3,4], and the market size of Inconel 718 may increase to over $5 billion in 2026 [5]. Although the machinability of Inconel 718 has been improved over the years, it remains a difficult-to-cut material due to its high temperature strength, high chemical affinity, low thermal conductivity, and strong work-hardening tendency. In addition, in response to climate change and its impacts, especially the global warming of 1.5 °C, the 17 Sustainable Development Goals (SDGs) were adopted by the United Nations in 2015, and carbon neutrality was subsequently proposed by the IPCC in 2018 [6,7]. The goal of carbon neutrality by 2050, according to the Paris Agreement, has been adopted and announced by many countries [8]. Achieving the SDGs and carbon neutrality has become the most urgent and important mission at present. Manufacturing, especially the machining process, as the most influential industry in the global economy, is one of the main causes of carbon emissions due to its massive energy and material consumption [9]. In other words, reducing the energy and material consumptions in machining Inconel 718 can greatly advance the process toward carbon neutrality. Therefore, there is an urgent need to develop a more sustainable and low-cost way for environmentally friendly machining Inconel 718.

The high-temperature strength and the low thermal conductivity are the main reasons why Inconel 718 is very difficult to cut [10]. Inconel 718 has a relatively high strength at room temperature and can still maintain a large portion of its mechanical properties with only a 20% drop at high temperatures up to 700 °C [11]. This results in the generation of large cutting forces during cutting, even at high cutting temperatures. The main cutting force generated when dry cutting Inconel 718 can be about 1.4 times larger than that obtained when dry cutting stainless steel SUS304 [12,13]. On the other hand, the low thermal conductivity can reduce the portion of the generated heat that is transported away with a chip or conducted to a workpiece, resulting in high heat accumulation in the cutting zone. Therefore, high cutting temperatures can occur even at low cutting speeds. Cutting temperatures can reach about 760 °C at cutting speeds of 30 m/min, and 1050 °C at 100 m/min [14]. Since the material properties of tool material such as hardness, strength, chemical stability, and wear resistance decrease with increasing temperature, which can result in severe tool wear and short tool life, the selection of cutting tool materials for cutting Inconel 718 is very important. In practice, there are only four suitable cutting tool materials for cutting Inconel 718. Uncoated cemented carbide tools are very suited for cutting Inconel 718 due to their low cost, high toughness, and high thermal conductivity. However, they are restricted to cutting at a cutting speed below 50 m/min because of their poor thermochemical stability [15]. PVD/CVD-coated cemented carbide tools with appropriate coatings can increase the recommended cutting speed up to 80 m/min [16]. However, under certain cutting conditions, they can lead to worse surface roughness [17] and cannot achieve a longer tool life than uncoated cemented carbide tools [12]. For high-speed cutting (exceeding 100 m/min), ceramic tools or cubic boron nitride (CBN) cutting tools should be used. Whisker-reinforced aluminum ceramic tools can improve the optimum cutting speed up to 250–310 m/min [18], and if used with the air-jet assisted method, the maximum cutting speed can reach up to 660 m/min [19]. However, ceramic tools are mostly limited to rough and semi-finish operations due to their spontaneous failure and less predictable tool life [20], and the tool life may be less than 1.5 min at cutting speeds above 250 m/min with the use of coolant [21]. CBN tools or new polycrystalline CBN (PCBN) tools are another possible alternative. It was reported that the maximum cutting speed of PCBN tools can reach 1250 m/min [22], and the tool life is about 5.8–8 min at 300 m/min with the use of coolant [23]. However, it should be noted that when dry cutting, the tool life is only about 0.2–1.6 min at 300 m/min [24]. Meanwhile, to obtain longer tool life and better product quality, various cutting fluid technologies [19,25,26] and tool improvement techniques (including tool geometries optimization technologies and tool texturing technologies [27,28], etc.) have also become at the forefront of Inconel 718 research and attracted attention in recent years.

According to the above-mentioned previous study, from a practical perspective, it is generally strongly recommended to use cutting fluid to improve the cutting efficiency of Inconel 718 in the industry. However, the use of conventional cutting fluid can not only cause several environmental pollutants and health and safety hazards but also increase the total cost of the manufacturing process [29,30]. With the growing awareness of sustainability issues, the applications of minimum quantity lubrication (MQL), high-pressure cooling (HPC), cryogenic lubrication, and the usage of bio-based cutting fluid (vegetable oil, coconut oil, etc.) have been developed in recent years, and these technologies have gained wide acceptance in the industry [29,30]. Moreover, the employment of a cooling and lubrication system increases the power consumption and the total manufacturing cost, since the cost for the cooling and lubrication system, including cutting fluids, equipment, power consumption, cleaning, etc., can take up to 10–17% of the total manufacturing cost [30]. Therefore, dry cutting is the best option to minimize the total manufacturing cost, reduce power and cooling oil consumption, and achieve environmentally friendly machining. At the same time, the deepening of our understanding of the dry cutting process can also bring significant technological changes to the tool material and cutting process.

In general, due to the characteristics of Inconel 718, intensive adhesive interaction occurs easily at the tool–workpiece interface, resulting in the built-up edge (BUE) or adhesion formation during cutting, which can cause poor surface quality, rapid tool wear, and tool chipping [16,22]. Meanwhile, since the adhesion is the accumulation of the work-hardened workpieces, its hardness is about 1.5–4 times higher than that of the workpiece [31,32]. The adhesion becomes the actual cutting tool surface and functions as a protective layer by separating the tool surface from the chip during cutting. Some studies have reported the protective effect of adhesion when cutting Inconel 718 [33,34]. From the point of view of taking full advantage of the adhesion, by actively and purposely utilizing adhesion, we can achieve the dry cutting of Inconel 718 more sustainably and economically. However, the understanding of the protective effect of adhesion is still not well established, and until now, no studies have given a way to utilize the adhesion in the dry cutting of Inconel 718.

To actively and purposely utilize adhesion, it is necessary to give a clear definition of adhesion. To the knowledge of the authors, the adhesion, strictly speaking, should be classified into three categories by its location and size: (1) built-up layer (BUL) on the tool rake face; (2) BUE around the cutting-edge; and (3) flank built-up (FBU) on the tool flank face [13,35]. BUE appears at low cutting speeds [35,36]. Because the BUE can grow to a noticeable size around the cutting-edge [36], it can change the shape of the cutting-edge and affect cutting forces and surface quality. Since the BUE is always unstable and experiences a periodic formation, growth, and fall-off process due to its large size, it is always associated with severe tool wear. The BUL is a thin and stable adhesion formed on the overall tool rake face when cutting carbon steel S45C and stainless steel SUS304 at high cutting speeds [35,36] or difficult-to-cut materials at low cutting speeds [37,38]. The BUL is shaped like a water drop with the highest point in the middle of the tool and chip contact zone, and its maximum height is always below 100 μm [36]. Due to this special shape, the BUL hardly causes the over-cutting phenomenon. And since its stability is very high, the BUL has few or no influences on the variation in cutting forces and surface quality. Since the BUL is also the accumulation of the work-hardened workpieces, it can also function as a protective layer during cutting [12,13,37,38]. However, as the cutting speed continues to increase, the stable BUL disappears, and only some thin layers or small pieces of adhesion can be sporadically formed on the tool surface, which is very unstable and can lead to adhesive wear. In addition, the FBU is the accumulation of workpieces on the tool flank face. According to this definition, the BUL should not be treated as a predecessor of the BUE, and the thin layer or small piece of adhesion should also not be treated as the BUL; these opinions differ from those in Refs. [16,22,33,34]. Considering the protective of the BUL, we can actively and purposely utilize the BUL to realize the self-protective tool (SPT) during cutting. This SPT method has been verified when dry cutting SUS304 [13]. In our previous studies, the authors investigated the influences of the BUL on the uncoated, PVD/CVD-coated cemented carbide tools when dry cutting Inconel 718 [12,15,39]. However, until now, due to the small height of BUL (<100 μm), the BUL formation phenomenon and the effectiveness of the SPT method in the dry cutting of Inconel 718 have not been fully understood and studied yet.

This paper proposed a novel approach, which can be called the SPT method, for dry cutting Inconel 718 in a more sustainable and low-cost way by actively and purposely utilizing the BUL. To verify this new approach, various cutting experiments were carried out using the aged-treated Inconel 718 and relatively inexpensive uncoated cemented carbide tools. The BUL formation phenomenon in the dry cutting of Inconel 718 were systematically investigated. Section 3 presents these results in three parts. The formation condition of the BUL, its formation mechanism, and its stability were discussed in Section 3.1. The influences of the BUL on cutting forces and surface roughness were investigated in Section 3.2. The protective effect of BUL was evaluated in detail in Section 3.3 and the FBU formation and its protective effect were also investigated. Section 4 offers a discussion on the effectiveness and generalizability of the proposed SPT method by comparing the results under different cutting atmospheres and on different material microstructures of Inconel 718. The conditions for realizing the SPT in the dry cutting of Inconel 718 are also proposed.

## 2. Experimental Section

### 2.1. Workpiece Material and Cutting Tools

**Figure 1:**
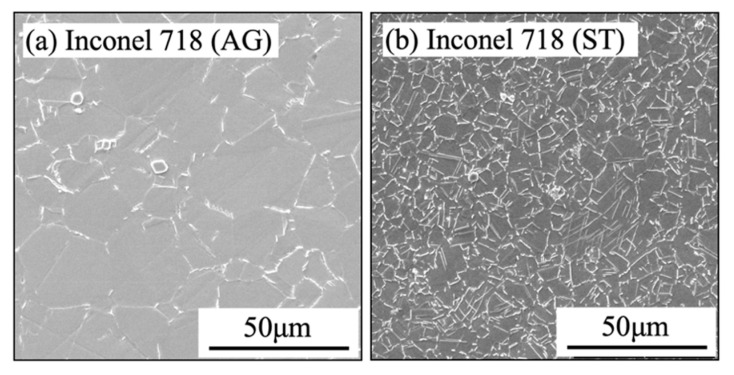
Microstructures of (**a**) Inconel 718(AG) and (**b**) Inconel 718(ST).

Commercially available age-treated Inconel 718 (Inconel 718(AG)) (982 °C, 1 h, water quench; precipitation heat treat 718 °C, 8 h; furnace cool to 621 °C, 8 h; air cool) was selected as workpiece material. To verify the generalizability of the proposed SPT method, a commercially available Inconel 718 in a solution-annealed treatment (Inconel 718(ST)) (955 °C, 1 h, water quench) was also used. Their chemical compositions and mechanical properties are listed in Table 1 and Table 2, respectively. The hardness was measured at room temperature using a micro-Vickers hardness tester (HMV1/2, Shimadzu, Kyoto, Japan). The microstructures of Inconel 718(AG) and Inconel 718(ST) are shown in Figure 1. Here, the average grain size was measured using the intercept standard for average grain size measurements. The average grain size of Inconel 718(AG) is 33 ± 14 μm, which is about 3.7 times larger than that of Inconel 718(ST) (9 ± 2 μm). To avoid any additional effects for each material (such as chemistry compositions, microstructure, and so on), all workpieces (200 mm in length, 45 mm in diameter) from the same bar were used.

To promote the BUL formation and to eliminate the effects of tool coatings on the BUL formation, uncoated cemented carbide inserts without chip breaker (rake angle 0°, flank angle 11°, cutting-edge radius 2.5 μm, and no honing, K10, TPGN160304, Tungaloy, Fukushima, Japan) were used in this study. The inserts were set on a tool holder that had a seating surface with a rake angle of 5° and a flank angle of 6° (CTGPR2020K3, Tungaloy, Fukushima, Japan). Therefore, the actual rake angle and flank angle after mounting were 5° and 6°, respectively. The tool’s nose radius was 0.4 mm. As the tool cutting-edge angle was 91°, the uncut chip thickness $t_{u}$ can be calculated as $t_{u}=f\;*\;sin(91{}^{\circ})\approx f$, and the uncut chip width b can be calculated as b=w/sin(91°)≈w in this study, where f is the feed rate and w is the cutting depth.

To investigate the influence of cutting-edge radius on the BUL formation, three different cutting-edge radii (2.5, 80, and 165 μm) were used in this study. The cutting-edge radii of 80 and 165 μm were prepared using a diamond flap wheel (grit size #400, diameter 15 mm, Ichiguchi, Kyoto, Japan), and the cutting-edge radius was controlled by changing the grinding speed, grinding time, and feed rate.

### 2.2. Experimental Set-Up and Analysis

A series of 3-D bar-turning tests were performed on a CNC lathe machine (MULTUS B200, Okuma, Aichi, Japan) to examine the BUL formation phenomenon in dry cutting of Inconel 718. As mentioned above, one purpose of this work is to verify the effectiveness of the SPT method. To verify this new approach, turning tests with cool air or cutting fluid were also performed. Cool air or cutting fluid was supplied to the cutting point through the machine’s coolant system, not through external equipment. Here, a commercially available water-soluble cutting fluid (Hi-chip NC-11, Taiyu, Osaka, Japan) diluted 10 times with water was used.

Cutting conditions were selected to cover data that were equal to or larger than those recommended by tool manufacturers. Five cutting speeds from 10 to 90 m/min, eight feed rates from 0.001 to 0.2 mm/rev, and three cutting depths of 0.5, 1, and 2 mm were used. Since the tool’s nose radius is 0.4 mm, the smallest cutting depth of 0.5 mm was selected. To study the BUL formation mechanism, cutting times of 1, 10, 30, 60, 120, … 300 s were used. The cutting conditions are listed in Table 3. The pre-machining (with a cutting speed of 10 m/min, a feed rate of 1 mm/rev, and a cutting depth of 1 mm) was performed before each turning test to remove the plastic deformation layer formed by the previous turning. Each test was replicated three times to maintain the repeatability of the cutting test.

**Figure 2:**
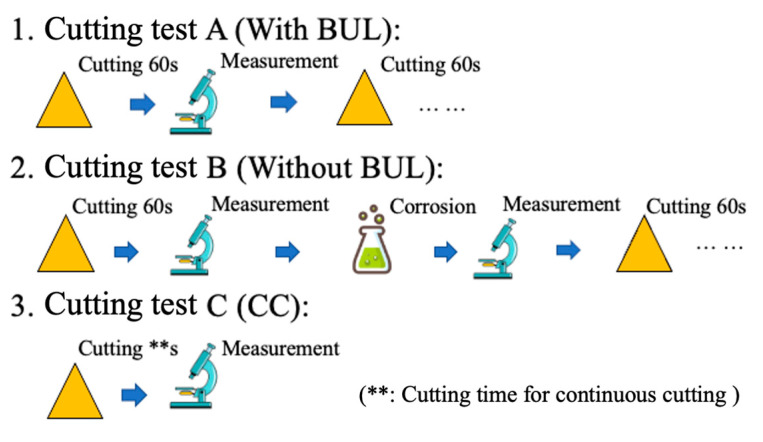
Schematic illustration of cutting tests for comparative experiment.

A comparative experiment was also carried out between the state of the cutting tool with and without a BUL after a period of cutting time to study the stability and protective effect of BULs. As shown in Figure 2, for the turning test A (With BUL), the tool was observed every 60 s of cutting without removing the BUL. For the turning test B (Without BUL), the tool was observed every 60 s of cutting, and the BUL was removed using corrosion before the next cutting. Turning test C (CC) represents continuous cutting without interruption. Here, the BUL was removed by placing the tool into Kalling’s No. 2 reagent (5 g CuCl_2_, 100 mL HCl, 100 mL ethanol) for about 5–8 min until the BUL was removed.

Cutting forces were measured using a three-component piezoelectric dynamometer (9129A, Kistler, Kanagawa, Japan) at a 1 kHz sampling rate. The finished surface was measured using a surface roughness tester (SJ-201, Mitutoyo, Kanagawa, Japan) with a cut-off wavelength of 0.8 mm and a filter of 2CR75. The surface roughness measurement was repeated four times (measuring points spaced at 90° intervals in the circumferential direction), and the average value was used for the analysis. The surface roughness parameters used in this study are the arithmetical mean surface roughness value (*Ra*) and the mean maximum height of the profile (*Rz*), which are generally used in the industry.

**Figure 3:**
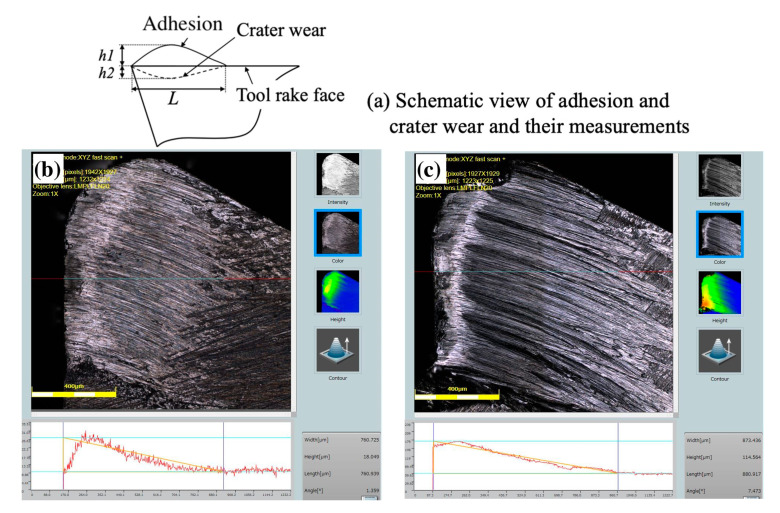
(**a**) Schematic view of adhesion and crater wear and their measurements, and typical LCM observation results on tool rake face after cutting 60 s in dry cutting of (**b**) Inconel 718(AG) and (**c**) Inconel 718(ST) at cutting speed 10 m/min, feed rate 0.1 mm/rev, cutting depth 1 mm.

The used tools after cutting were examined in detail using a scanning electron microscope (SEM, Quanta250, FEI, Tokyo, Japan), and laser confocal microscopy (LCM, OLS 4100, Olympus, Tokyo, Japan, and VK-200, Keyence, Osaka, Japan) to study the BUL formation process and to measure the tool wear. The mean tool flank wear (*VB*) was determined by measuring the tool flank wear land width ten times at equal intervals from the tool tip to the cutting depth. The notch wear, which appears at the cutting depth, was measured separately and is reported as its maximum value. The tool life criteria used in this study is an average width of the flank wear of *VB* = 300 μm according to ISO 3685:1993. As shown in Figure 3a, the mean maximum height of adhesion (including BUL and BUE) above the tool rake face *h*_1_, mean contact length between the adhesion and the cutting tool *L*, and the mean maximum depth of crater wear below the tool rake face *h*_2_ were measured using LCM, and their mean values were also determined by measuring ten times at equal intervals from the tool tip to the cutting depth. For example, the representative observation results of the tool rake face in the dry cutting of Inconel 718(AG) and Inconel 718(ST) at a cutting speed of 10 m/min, a feed rate of 0.1 mm/rev, a cutting depth of 1 mm, and a cutting time of 60 s are shown in Figure 3b,c. As shown, the morphology of adhesion for Inconel 718(AG) is completely different from that for Inconel 718(ST), which indicates that the material microstructure can have a significant influence on the morphology of adhesion. We will discuss these in Section 4.

## 3. Results and Discussion

### 3.1. Investigation of BUL Formation during Cutting Inconel 718(AG)

In the first part of the results, the influences of cutting conditions (cutting-edge radius, cutting speed, feed rate, cutting depth, and cutting atmosphere) on the BUL formation condition, the morphology of the BUL, the formation mechanism of the BUL, and the stability of the BUL are investigated in detail.

#### 3.1.1. Influence of Cutting-Edge Radius

**Figure 4:**
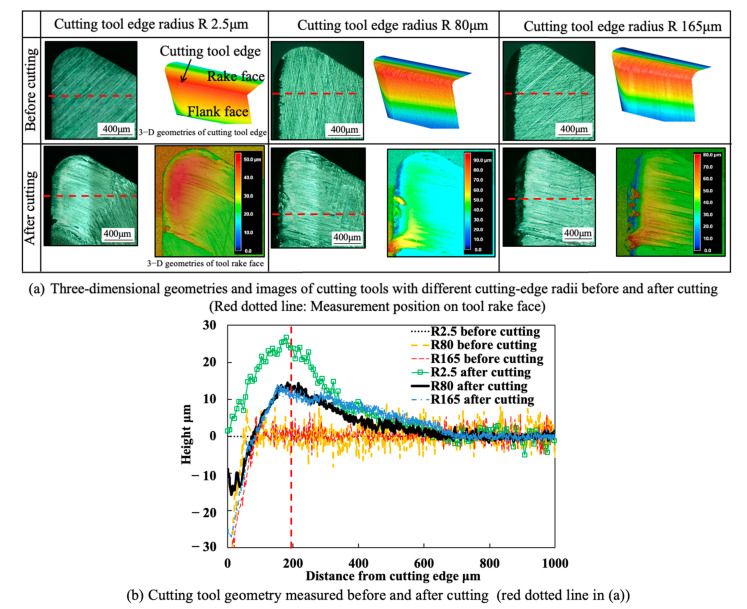
(**a**) Three-dimensional geometries and images of cutting tools with different cutting-edge radii before and after cutting, and (**b**) corresponding cutting tool geometry measured before and after cutting (red dotted line in (**a**)) in dry cutting of Inconel 718(AG) at cutting speed 10 m/min, feed rate 0.1 mm/rev, cutting depth 1 mm, cutting time 60 s.

The cutting-edge radius, which is the wedge shape formed by the transition from the rake face to the flank face, can significantly affect cutting phenomena, such as BUL formation, chip formation, contact condition, friction, and so on. Meanwhile, since the cutting-edge radius can change along the cutting process, it may affect the BUL formation after a certain period of cutting time. In our previous study, it was also found that the cutting-edge radius under a state of equilibrium changes with the cutting condition [41]. For these reasons, the influence of the cutting-edge radius on the BUL formation was first investigated. According to the results of previous studies [12,15], a cutting speed of 10 m/min, a feed rate of 0.1 mm/rev, a cutting depth of 1 mm, and a cutting time of 60 s were selected as the point at which the highest BUL could be formed in the dry cutting of Inconel 718(AG). Figure 4 shows the 3-D geometries and images of cutting tools with different cutting-edge radii (2.5, 80, and 165 μm) before and after cutting and the corresponding BULs’ profiles. As shown, the BUL, which resembles a water drop, forms thinly on the tool rake face and develops its highest point in the middle of the tool and chip contact zone in all cases. This is also the distinctive characteristic that distinguishes BULs from BUEs, which can grow up to a noticeable size around the cutting-edge [13]. On the other hand, as shown, although the maximum height of a BUL for the large cutting-edge radius (80 and 165 μm) is smaller than that for the small cutting-edge radius of 2.5 μm, a noticeable BUL can also be formed on the tool. And the position of the peak of the BUL for the large cutting-edge radius is almost the same as that for the small cutting-edge radius. These results indicate that the cutting-edge radius has few or no influences on the BUL formation and the position of the peak of the BUL, except for the height of the BUL. This result also indicates from another direction that the accumulated material of the BUL comes from the undersurface of the chip rather than the workpiece material around the cutting tool edge; this perspective differs from that of the BUE formation in Ref. [42]. In addition, since the BUL can remain on the tool rake face even when the tool’s cutting-edge changes greatly, it can be said that the BUL can be used to realize the STP during cutting. Based on these results, we will mainly discuss the BUL phenomenon when using a cutting tool with a cutting-edge radius of 2.5 μm in this study.

#### 3.1.2. Influence of Cutting Speed

**Figure 5:**
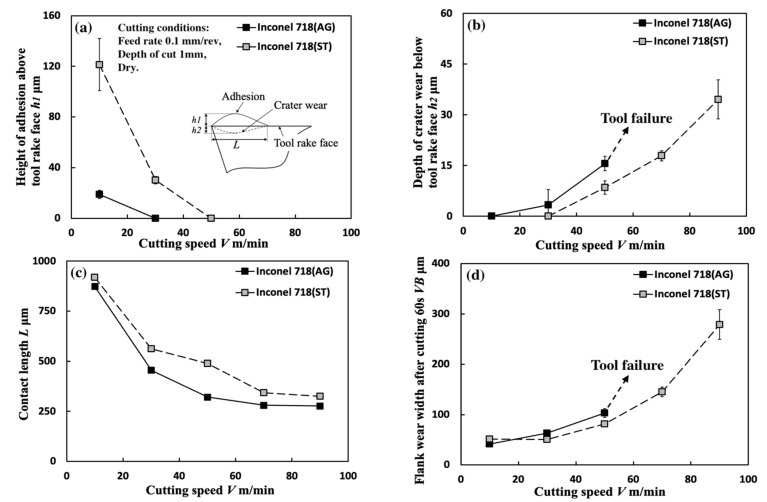
Variations in (**a**) maximum height of adhesion (including BUL and BUE) above tool rake face *h*_1_, (**b**) maximum depth of crater wear below tool rake face *h*_2_, (**c**) contact length between adhesion and cutting tool *L*, and (**d**) tool flank wear width *VB* with cutting speed in dry cutting of Inconel 718(AG) and Inconel 718(ST) with feed rate 0.1 mm/rev, cutting depth 1 mm, cutting time 60 s.

**Figure 6:**
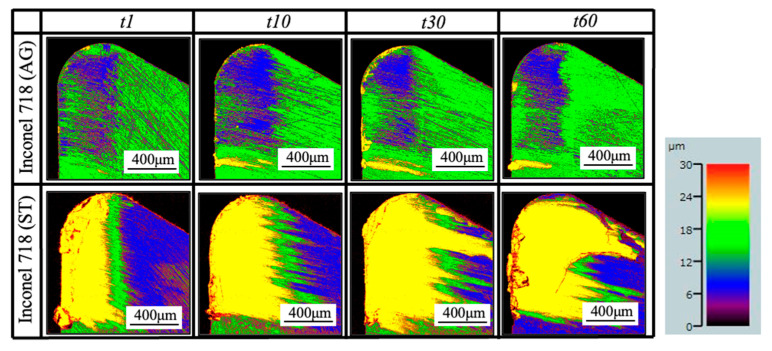
Three-dimensional geometries of tool rake face after cutting 1, 10, 30, and 60 s in dry cutting of Inconel 718(AG) and Inconel718(ST) at cutting speed 30 m/min, feed rate 0.1 mm/rev, cutting depth 1 mm.

**Figure 7:**
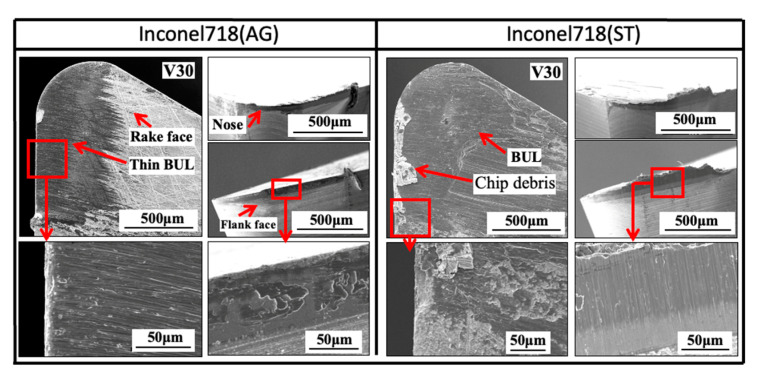
SEM images of cutting tool after cutting 60 s in dry cutting of Inconel 718(AG) and Inconel 718(ST) at cutting speed 30 m/min, feed rate 0.1 mm/rev, cutting depth 1 mm.

Figure 5 shows the variations in *h*_1_, *h*_2_, *L*, and *VB* with cutting speed in dry cutting of Inconel 718(AG) with a feed rate of 0.1 mm/rev, a cutting depth of 1 mm, and a cutting time of 60 s. As a comparison, the results of dry cutting Inconel 718(ST) are also presented. As shown, according to the results of previous studies [12,15], the adhesion formed on the tool rake face should be called the BUL at a cutting speed of 10 m/min. And it can be clearly seen that the cutting speed exerts significant influences on the size and formation condition of the BUL. As shown in Figure 5a, the highest BUL appears at a low cutting speed 10 m/min, and its maximum height *h*_1_ decreases as the cutting speed increases. And *h*_1_ becomes zero, and crater wear appears on the tool rake face when the cutting speed is up to 30 m/min (Figure 5b). On the other hand, *L* also decreases as the cutting speed increases (Figure 5c). To confirm the BUL formation at 30 m/min, the tool surface after cutting for 60 s was observed in more detail. Figure 6 shows the 3-D geometries of the tool rake face for four different cutting times (1, 10, 30, and 60 s), and Figure 7 shows the SEM images of the tool surface after cutting 60 s at 30 m/min. As shown, although the tool crater wear occurs quickly after cutting 1 s at 30 m/min, a very thin BUL with a height below the tool rake face can also form on the worn tool rake face. And the crater wear area has a decreasing trend with the cutting time, which indicates the workpiece material is accumulating on the tool rake face and the thin BUL is growing. Since the cutting temperature can rise to 760 °C at 30 m/min in the dry cutting of Inconel 718 [14], these phenomena can be explained as the result of the sharp increase in ductility and decrease in the work hardening rate of Inconel 718 when the temperature exceeds approximately 700 °C [11] and the pronounced drop in the hardness of WC-Co carbide metal above 700–800 °C [43]. In addition, this result also indicates that the thin BUL formed at 30 m/min can work as a protective layer and prevent the tool from wearing, which agrees with the results reported by ref. [34]. The authors of ref. [34] also confirmed the protective effect of thin adhesion when cutting the aged Inconel 718 at cutting speeds above 30 m/min. Due to the higher cutting temperature at cutting speeds above 50 m/min, the thin BUL disappears, and tool crater wear increases quickly (Figure 5b). For *VB* after cutting 60 s, *VB* increases as the cutting speed increases, and the tool life is less than 60 s when the cutting speed increases above 70 m/min (Figure 5d).

Based on these results, the BUL formation condition in the dry cutting of Inconel 718(AG) with a feed rate of 0.1 mm/rev and a cutting depth of 1 mm is given below: only BUL can be formed in the cutting speed range below 30 m/min. Meanwhile, as shown in Figure 5, Figure 6 and Figure 7, similar results can also be found in the dry cutting of Inconel 718(ST), but it seems that the material microstructure can significantly affect the formation condition of BUL. We will discuss these in Section 4.

#### 3.1.3. Influence of Feed Rate

**Figure 8:**
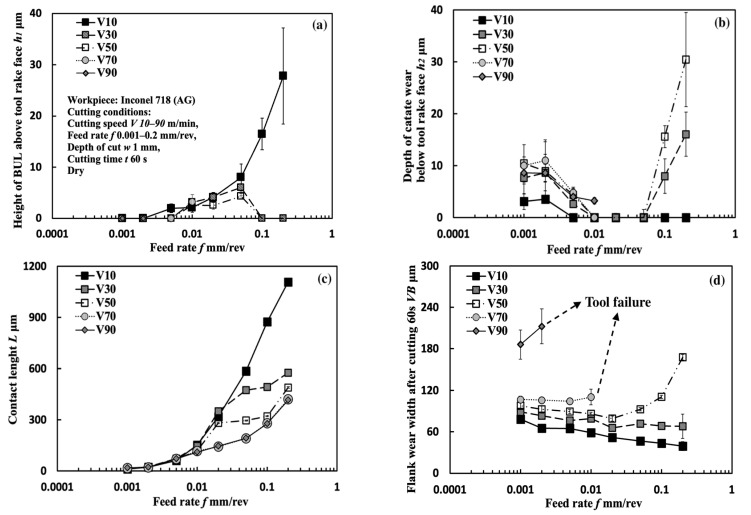
Variations in (**a**) maximum height of BUL above tool rake face *h*_1_, (**b**) maximum depth of crater wear below the tool rake face *h*_2_, (**c**) contact length between adhesion and cutting tool *L*, and (**d**) tool flank wear width *VB* with feed rate in dry cutting of Inconel 718(AG) at cutting speeds 10, 30, 50, 70, and 90 m/min, with cutting depth 1 mm, cutting time 60 s.

**Figure 9:**
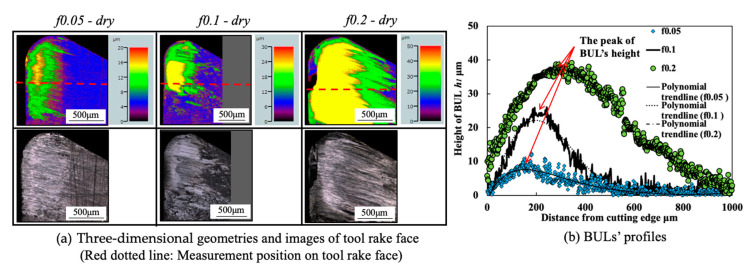
(**a**) Three-dimensional geometries and images of tool rake face, and (**b**) corresponding BULs’ profiles (red dotted line in (**a**)) in dry cutting of Inconel 718(AG) at cutting speed 10 m/min, feed rate 0.05, 0.1, and 0.2 mm/rev, cutting depth 1 mm, cutting time 60 s.

Figure 8 shows the variations in *h*_1_, *h*_2_, *L*, and *VB* with a feed rate range of 0.001–0.2 mm/rev under different cutting speeds in the dry cutting of Inconel 718(AG) with a cutting depth of 1 mm and a cutting time of 60 s. To confirm the morphology of the BUL, the BULs’ profiles after cutting 60 s were also investigated. For example, the results at a cutting speed of 10 m/min and feed rates of 0.05 and 0.2 mm/rev are shown in Figure 9. For comparison, the results at a feed rate of 0.1 mm/rev are also presented. As shown, according to the definition of the BUL [36], it can be said that only the BUL can be formed in the dry cutting of Inconel 718(AG) with different feed rates. And as expected, not only cutting speed but also feed rate have an overall strong influence on the size of the BUL. As shown in Figure 8a, it can be found that at 10 m/min, *h_1_* increases as the feed rate increases, and the highest BUL (with an average maximum height ~27.8 μm) forms at a feed rate of 0.2 mm/rev. At 30 and 50 m/min, a BUL with a height above the tool rake face can only occur in the feed rate range of 0.01–0.05 mm/rev. *h*_1_ increases as the feed rate increases, but it decreases as the cutting speed increases. When the cutting speed increases above 70 m/min, the BUL with a height above the tool rake face can only occur at 70 m/min with a feed rate of 0.01 mm/rev. These phenomena can be explained as the results of the increase in the thickness of the secondary shear zone (SSZ) at a large feed rate (i.e., uncut chip thickness $t_{u}$) and the temperature dependence of the strain rate hardening behavior of Inconel 718, which determines the adhesion formation [44]. Since the chip thickness $t_{c}$ increases as the feed rate increases, the thickness of SSZ $t_{SSZ}$ ($=\delta t_{c}$, with a material constant 0≤δ<1[45]) increases, but the average shear strain rate at the tool–chip interface ${\dot{\gamma }}_{int}$ (${\dot{\gamma }}_{int}=V_{c}/t_{SSZ},$ with the chip sliding speed $V_{c}=t_{u}V/t_{c}$, and chip thickness $t_{c}$ [45]) decreases as the feed rate increases. Since the accumulated material of the BUL comes from the deformed material in SSZ, as discussed above and in Ref. [36], it can be concluded that *h*_1_ increases as the feed rate increases. On the other hand, the work hardening of Inconel 718 decreases as the temperature increases or the strain rate decreases in the high temperature domain (>700 °C) [11]. Due to the high temperature and the decrease in strain rate at a large feed rate, which can result in the reduction in the work hardening of Inconel 718(AG), the BUL with a height above the tool rake face can only form at the smaller feed rate (<0.1 mm/rev) when the cutting speed is higher than 30 m/min. These results indicate that, compared with its influence on the BUL formation condition, the feed rate can play a more important role in the height of BUL *h*_1_. It can also be concluded that the BUL does not occur at the end of cutting but occurs during cutting since the feed decreases to 0 at the end of cutting.

The results shown in Figure 8b clarify that when the feed rate is below 0.01 mm/rev, tool crater wear can occur at each cutting speed, and *h*_2_ decreases as the feed rate increases. For the larger feed rate, tool crater wear occurs only when the feed rate is increased above 0.1 mm/rev and the cutting speed is above 30 m/min, and *h*_2_ increases as the feed rate increases. However, due to the short tool life, there are no data for *h*_2_ at 70 and 90 m/min when the feed rate exceeds 0.01 mm/rev. This variation trend of *h*_2_ can be explained as the result of the protective effect of the BUL since, as mentioned above, a higher BUL can form as the feed rate increases. It should be noted that, due to the severe crater wear, a BUL with a height higher than the tool rake face cannot be observed under certain feed rates.

The results shown in Figure 8c clarify that *L* increases as the feed rate increases, and the cutting speed can affect *L* only when the feed rate exceeds 0.02 mm/rev. These results indicate that the feed rate can also play an important role in *L*.

It is generally known that *VB* increases as the feed rate increases [46,47], because the cutting temperature increases as the feed rate increases. However, as shown in Figure 8d, this tendency was only confirmed at high cutting speeds above 50 m/min with larger feed rates. In contrast, *VB* decreases noticeably with the increase in feed rate at 10 and 30 m/min, 50 m/min in the feed rate range of 0.001–0.02 mm/rev, and 70 m/min in the feed rate range of 0.001–0.005 mm/rev. These results indicate that BULs protect the tool from further wear.

Additionally, as shown in Figure 9, it is worth stating that the peak of the BUL is almost in the middle of the tool and chip contact zone, and the distance from the cutting edge to the peak of the BUL is larger than the feed rate, and its value increases as the feed rate increases. These features confirm that the feed rate can also play a very important role in the BUL formation process.

#### 3.1.4. Influence of Cutting Depth

**Figure 10:**
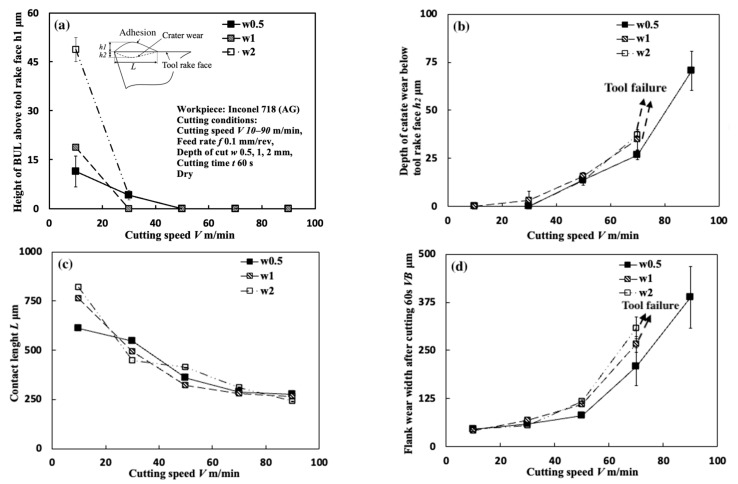
Variations in (**a**) maximum height of BUL above tool rake face *h_1_*, (**b**) maximum depth of crater wear below the tool rake face *h*_2_, (**c**) contact length between adhesion and cutting tool *L*, and (**d**) tool flank wear width *VB* with cutting speeds at different cutting depths in dry cutting of Inconel 718(AG) with feed rate 1 mm/rev, cutting time 60 s.

**Figure 11:**
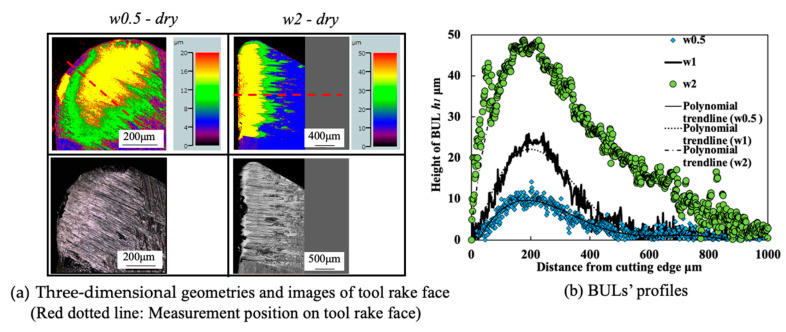
(**a**) Three-dimensional geometries and images of tool rake face, and (**b**) corresponding BULs’ profiles (red dotted line in (**a**)) in dry cutting of Inconel 718(AG) at cutting speed 10 m/min, feed rate 0.1 mm/rev, cutting depths 0.5, 1, and 2 mm, cutting time 60 s.

Figure 10 shows the variations in *h*_1_, *h*_2_, *L*, and *VB* with cutting speeds at three different cutting depths in the dry cutting of Inconel 718(AG) with a feed rate of 0.1 mm/rev and a cutting time of 60 s. Figure 11 shows the BUL’s profile at a cutting speed of 10 m/min. For comparison, the results at a cutting depth of 1 mm are also presented. As shown, according to the definition of the BUL [36], it can be said that a BUL can be formed in the cutting speed range below 50 m/min at cutting depths of 0.5 and 2 mm, and this formation cutting speed range of BULs is larger than that obtained at a cutting depth of 1 mm. It can also be seen in Figure 10a,c that the cutting depth can give a strong influence on the size of the BUL only at 10 m/min. Both the height of the BUL and its contact length increase as the cutting depth increases. However, the influence of the cutting depth becomes smaller as the cutting speed increases to 30 m/min. At cutting speeds above 50 m/min, a BUL with a height above the tool rake face cannot be formed in all cases. This changing trend in the BUL’s size at different cutting depths can be explained as the result of an increase in the thickness of the chip. To prove this explanation, the chip thicknesses for three cutting depths were measured. It was found that at 10 m/min, the chip thickness $t_{c}$ at a cutting depth of 2 mm ($t_{c}$: 224.375±18.083 μm) is about 9.5% larger than that obtained at a cutting depth of 1 mm ($t_{c}$: 204.875±7.765 μm), and about 36.0% larger than that obtained at a cutting depth of 0.5 mm ($t_{c}$: 165.000±27.212 μm). These changes in the thickness of the chip contribute to a thick SSZ, resulting in a larger BUL. On the other hand, as the cutting speed increases above 30 m/min, the influence of the cutting depth on the cutting temperature increases. It was found that at 30 m/min, the chip thickness $t_{c}$ at a cutting depth of 2 mm ($t_{c}$: 202.750±7.232 μm) is about 5.5% larger than that obtained at a cutting depth of 1 mm ($t_{c}$: 192.00±10.607 μm), and about 24.3% larger than that obtained at a cutting depth of 0.5 mm ($t_{c}$: 163.125±19.553 μm). However, as discussed in Section 3.1.3, the high temperatures above 700 °C can lead to an increase in the ductility and reduction in the strain rate hardening rate of Inconel 718, which inhibit the BUL formation. Therefore, under their interaction, a BUL with a height above the tool rake face can only be formed at cutting depths of 0.5 and 2 mm with a cutting speed of 30 m/min.

Meanwhile, it is generally known that tool wear increases as the cutting depth increases [45,46]. However, as shown in Figure 10b,d, comparing with the results at cutting speeds above 50 m/min, the cutting depth has few influences on the depth of crater wear and the width of flank wear at 10 and 30 m/min. These results indicate that at different feed rates, the BUL can give a protective effect and protect the tool from further wear.

It can also be confirmed in Figure 11 that the cutting depth has almost no influence on the morphology of BUL or the distance from the cutting edge to the peak of BUL. Since the cutting temperature at 10 m/min in the dry cutting of Inconel 718 is only about 400–500 °C [14,48], the influence of strain rate on the material strength of Inconel 718 can be neglected [11]. And the influence of cutting depth on cutting temperature can also be neglected at the low cutting speed of 10 m/min [32]. Therefore, the hardening and fracture behaviors of Inconel 718 at different cutting depths are relatively close. As a result, BUL presents a very close result for different cutting depths.

#### 3.1.5. Influence of Cutting Atmosphere

**Figure 12:**
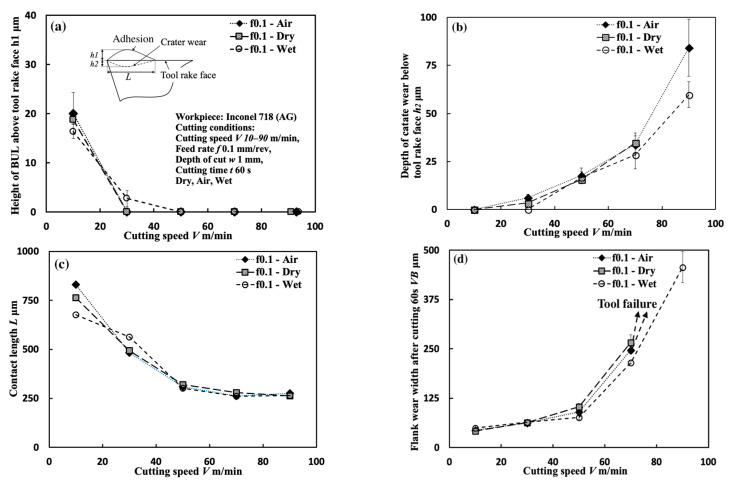
Variations in (**a**) maximum height of BUL above tool rake face *h*_1_, (**b**) maximum depth of crater wear below the tool rake face *h*_2_, (**c**) contact length between adhesion and cutting tool *L*, and (**d**) tool flank wear width *VB* with cutting speed under different cutting atmospheres (dry, air, and wet) in cutting of Inconel 718(AG) with feed rate 1 mm/rev, cutting depth 1 mm, cutting time 60 s.

**Figure 13:**
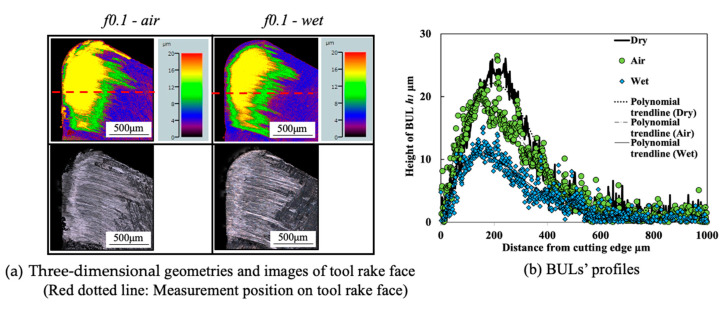
(**a**) Three-dimensional geometries and images of tool rake face, and (**b**) corresponding BULs’ profiles (red dotted line in (**a**)) in cutting of Inconel 718(AG) under air and wet cutting atmospheres at cutting speed 10 m/min, feed rate 0.1 mm/rev, cutting depth 1 mm, cutting time 60 s.

**Figure 14:**
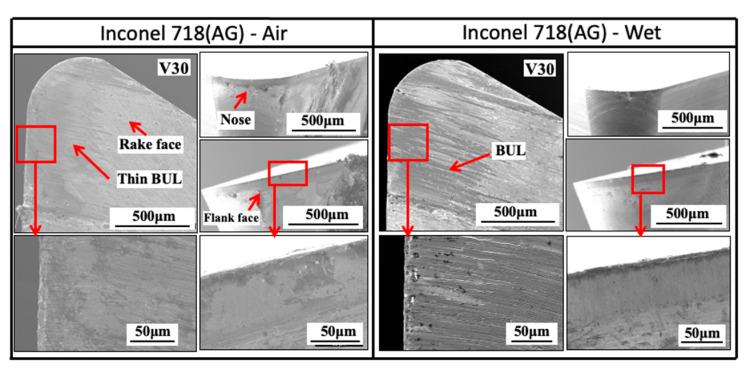
SEM images of cutting tool after cutting 60 s in cutting of Inconel 718(AG) under air and wet atmospheres at cutting speed 30 m/min, feed rate 0.1 mm/rev, cutting depth 1 mm.

To evaluate the protective effect of BUL in dry cutting, it is also necessary to investigate the BUL formation phenomenon under different cutting atmospheres. For this reason, the formation conditions of BUL under air and wet cutting atmospheres were also investigated in detail. Figure 12 shows the experimental results of *h*_1_, *h*_2_, *L*, and *VB* after cutting 60 s under air and wet cutting atmospheres. Figure 13 shows the BUL’s profile at a cutting speed of 10 m/min. For comparison, the results under the dry cutting atmosphere are also presented. As shown, according to the definition of BUL [36], it can be said that only BUL can be formed on the tool rake face in the cutting speed range below 50 m/min in air and wet cuttings of Inconel 718(AG), and this BUL formation cutting speed range is larger than that obtained when dry cutting. It can also be seen that the changing trends of *h*_1_, *h*_2_, *L*, and *VB* are almost the same under different cutting atmospheres, which indicates that the three different cutting atmospheres have similar influences on the BUL’s formation and tool wear formation. However, the air and wet cutting atmospheres can slightly affect the size of the BUL at a low cutting speed below 30 m/min and reduce tool wear at a high cutting speed above 50 m/min. At 10 m/min, the air cutting atmosphere tends to increase the height and contact length of the BUL, while the wet cutting atmosphere tends to decrease them compared with the results of the dry cutting atmosphere. However, as the cutting speed increases above 30 m/min, except in the wet atmosphere, tool crater wear occurs on the tool rake face, and only the thin BUL with a height below the tool rake face can be formed (Figure 7 and Figure 14). It can also be confirmed in Figure 14 that the wet cutting atmosphere can still ensure the formation of BUL without crater wear at 30 m/min. These results indicate that neither the cool air nor the cutting fluid can penetrate the contact region between the tool and chip near the cutting edge during cutting, thus ensuring the necessary contact condition for the BUL formation. At 10 m/min, since the air cutting atmosphere can reduce the cutting temperature around the cutting edge, a relatively larger BUL can be formed. The cutting fluid can reduce the cutting temperature, but it also has a lubricating effect at the same time. Therefore, it can penetrate deeper into the tool and chip contact region and affect the friction at the tool–chip interface, eventually leading to a reduction in the size of the BUL. In addition, due to the higher cooling effect of cutting fluid, the wet cutting atmosphere has a lower cutting temperature than the other two cutting atmospheres at 30 m/min, which promotes the accumulation of workpiece material on the tool and prevents the decrease in the hardness of WC-Co carbide metal. Thus, the BUL with a height above the tool rake face occurs at 30 m/min in wet cutting.

As shown in Figure 12d, it can be found that *VB* has a quickly increasing trend as the cutting speed increases above 50 m/min, and its value can reach above 200 μm at 70 m/min in all cases. These results confirm that Inconel 718 is difficult to cut at cutting speeds above 50 m/min even using the machine’s coolant system, which also fully justifies the need for a detailed study of dry cutting Inconel 718. Additionally, it is worth stating that in the BUL formation cutting speed range of 10–30 m/min, the cutting atmospheres have few or no influences on both the depth of crater wear and the width of flank wear (Figure 12b,d). These results demonstrate that the BUL has a protective effect and can prevent the tool from further wear under different cutting atmospheres.

Meanwhile, as shown in Figure 13, the cutting atmosphere can slightly change the height of the BUL and the distance from the cutting edge to the peak of the BUL, but it has few influences on the morphology of the BUL. Therefore, the BUL formed under different cutting atmospheres can exhibit similar protective effects as discussed above.

**Figure 15:**
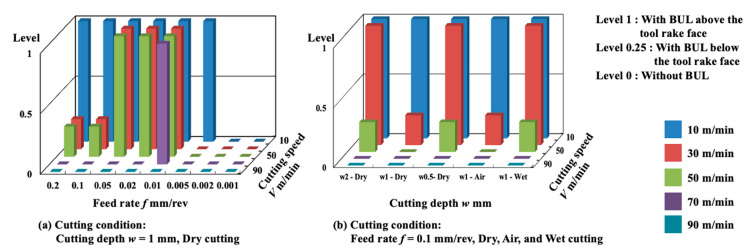
Measured BUL formation conditions in cutting of Inconel 718(AG).

According to the experimental results above, the BUL formation conditions during the cutting of Inconel 718(AG) are shown in Figure 15. It can be concluded that the cutting speed plays a decisive role in the BUL formation condition. In most cases, the BUL only occurs in the cutting speed range of ~30 m/min, and this cutting speed range can increase to ~50 m/min or decrease to ~10 m/min depending on the cutting conditions such as feed rate, cutting depth, and cutting atmosphere. The feed rate has a more significant influence on the BUL formation condition compared with cutting depth and cutting atmosphere. These results also confirm that the cutting conditions, such as cutting speed, feed rate, and cutting depth, can also play important roles in the height and length of the BUL in dry cutting Inconel 718(AG), which indicates that we can use the cutting conditions to control the size of the BUL and finally control its protective effect in dry cutting Inconel 718(AG).

#### 3.1.6. In-Detail Evaluation of BUL’s Formation Mechanism

**Figure 16:**
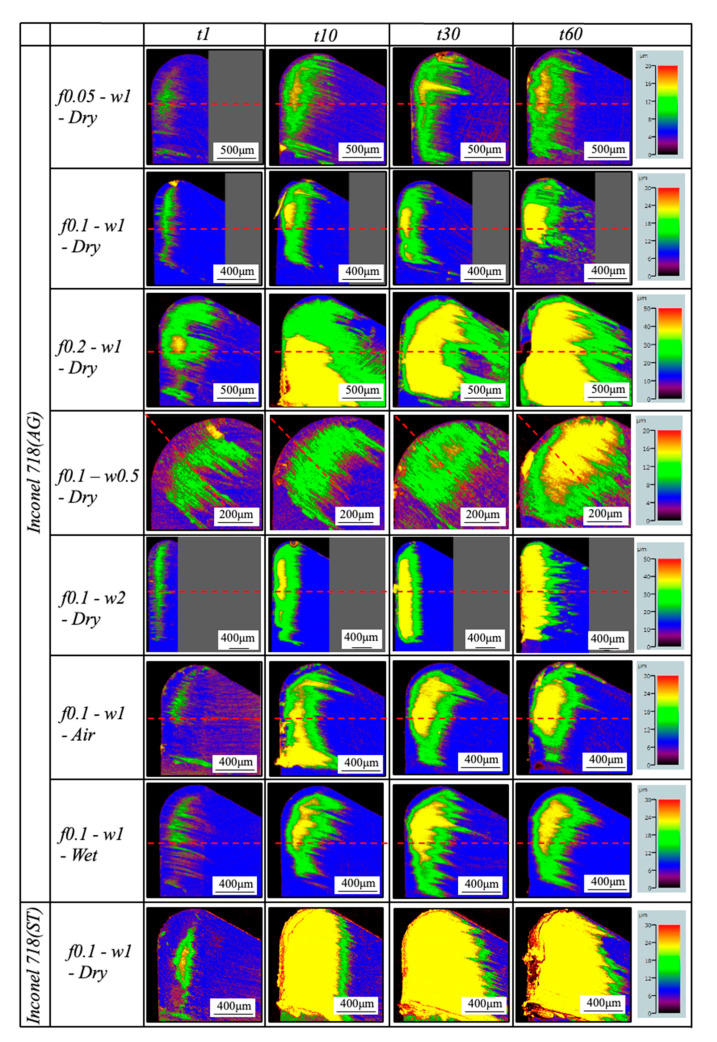
Three-dimensional geometries of tool rake face at cutting speed 10 m/min after cutting 1, 10, 30, and 60 s in cutting of Inconel 718(AG) and Inconel 718(ST) under different cutting conditions (feed rates: 0.05, 0.1, and 0.2 mm/rev, cutting depths: 0.5, 1, and 2 mm, cutting atmospheres: dry, air, and wet) (Red dotted line: measurement position on tool rake face).

**Figure 17:**
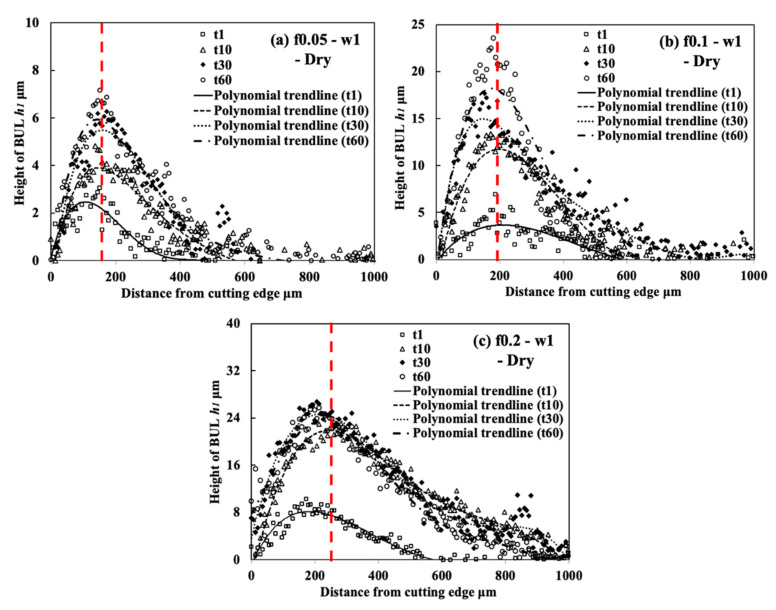
Variations in BULs’ profiles (red dotted line in Figure 16) with cutting time in dry cutting of Inconel 718(AG) at cutting speed 10 m/min, feed rates 0.05, 0.1 and 0.2 mm/rev, cutting depth 1 mm.

**Figure 18:**
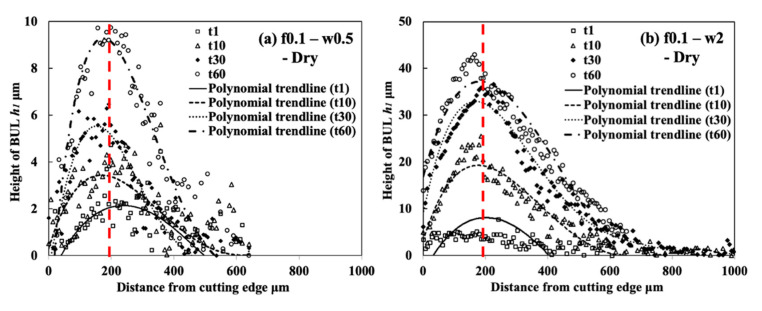
Variation in BULs’ profiles (red dotted line in Figure 16) with cutting time in dry cutting of Inconel 718(AG) at cutting speed 10 m/min, feed rate 0.1 mm/rev, cutting depths 0.5 and 2 mm.

**Figure 19:**
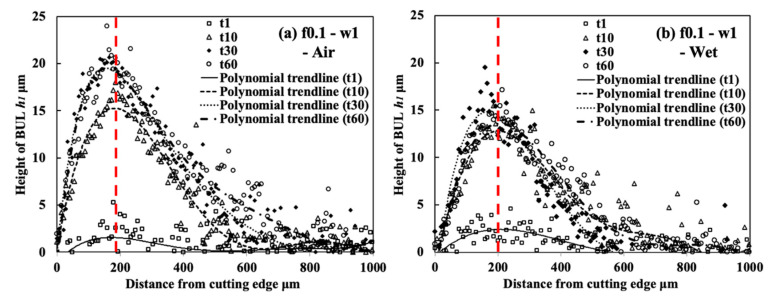
Variation in BULs’ profiles (red dotted line in Figure 16) with cutting time in air and wet cutting of Inconel 718(AG) at cutting speed 10 m/min, feed rate 0.1 mm/rev, cutting depth 1 mm.

Considering the BUL formation conditions discussed above, LSM analysis was applied to gain a deeper insight into the BUL formation mechanism under different cutting conditions. For illustration, the 3-D geometries of the tool rake face for four different cutting times (1, 10, 30, and 60 s) at a cutting speed of 10 m/min under different cutting conditions are shown in Figure 16. The corresponding variations in the BUL’s profile with cutting time are shown in Figure 17, Figure 18 and Figure 19. For comparison, the results in air and wet cutting of Inconel 718(AG) and dry cutting of Inconel 718(ST) are also presented.

As shown, it can be verified that the BUL can be treated as the result of the accumulation of the workpiece material from the chip undersurface onto the tool rake face, and the formation of BUL is a time-dependent phenomenon, which has also been discovered in the dry cutting of carbon steel S45C, hardened steel S45C, stainless steel SUS304, etc. [13,35,37,38]. It can also be found that not only the feed rate [15,39] but also the cutting depth and the cutting atmosphere have few or no influences on the BUL formation mechanism, but they can affect the height or contact length of the BUL. The BUL formation process can be summarized as follows: At cutting time 1 s, the initial BUL (its height is only about 2–8 μm) quickly occurs in the middle of the tool and chip contact zone at an appreciable distance from the cutting edge. This process is called the nucleus formation process [49]. After the initial BUL is formed, the BUL grows in the horizontal and vertical directions of the tool rake face as a result of the accumulation of the workpiece material. As a result, the peak of the BUL moves from the middle of the tool and chip contact zone to the cutting edge. This process is called the forward growing process [36]. As the cutting time increases to 30–60 s, the BULs’ growth stops. The reason for this is that there is a balance between the accumulation and wear (or squeeze-out) of material on the upper surface of the BUL, which determines the morphology of the BUL. This is the BUL formation mechanism. As shown, since the height of the BUL increases with the cutting time, it can be concluded that the BUL is very stable and no overall fall-off phenomenon occurs after its initial formation. Similar results can also be confirmed in the dry cutting of Inconel 718(ST) (Figure 16), which verifies the generality of the proposed formation mechanism. However, because the workpiece material accumulates on the tool surface quickly and, finally, a large adhesion can be formed on the tool rake face after cutting 10 s, it is difficult to identify the formation process directly from the experiments.

For the identified BUL formation mechanism, it is very important to study the growth rate of the BUL, which can affect not only the size of the BUL but also its influence on the cutting process. As shown in Figure 17, Figure 18 and Figure 19, the feed rate, cutting depth, and cutting atmosphere can significantly affect not only the maximum height of the BUL at 60 s but also the growth rate of the BUL in each formation process. The larger feed rate or larger cutting depth may accelerate the growth rate of the BUL within 30 s (Figure 17 and Figure 18), whereas the cooling cutting atmosphere may only accelerate the growth rate of the BUL within 10 s (Figure 19). The former may be due to the fact that a larger feed rate or cutting depth can increase the thickness of SSZ in the chip and cause the temperature at the tool–chip interface to reach its steady-state value rapidly. While the latter may simply be due to the fact that the cooling cutting atmosphere can cause the temperature to reach its steady-state value rapidly since the chip’s thickness $t_{c}$ at 10 m/min under different cutting atmospheres (dry: 204.875±7.765 μm, air: 214.875±5.595 μm, and wet: 211.500±5.983 μm) was found to be very close. In addition, it should be noted that the cutting speed can also have a great impact on the growth rate of BUL since it can significantly affect the cutting process.

Another very important point is what factors can affect the position of the initial BUL, the position of the BUL’s peak, and the morphology of the BUL. As shown, it can be found that in all cases, the initial BUL is located in the middle of the tool and chip contact zone, and the BUL is shaped like a water drop with the highest point in the middle of the tool and chip contact zone. If $L_{high}=\beta \cdot f$ is used to represent the relationship between the distance from the cutting edge to the peak of the BUL $L_{high}$ and feed rate f, it can be found that the coefficient β is larger than 1 and decreases as the feed rate increases. However, the cutting depth and cutting atmosphere have almost no influence on it. This result indicates the feed rate can play an important role in the position of the initial BUL and the position of the peak of the BUL. From the previous studies [36,49], it can be said that the position of the initial BUL is determined by the internal fracture on the chip undersurface and the adhesion phenomenon and friction at the tool and chip interface, while the position of the peak of the BUL is determined by the wear property of the BUL’s material and the adhesion phenomenon and friction at the chip and BUL interface. It has also been proven experimentally and theoretically that these positions must be at a point that is larger than or equal to the feed [36]. Because a larger feed rate can lead to more severe contact conditions and a larger frictional force at the tool and chip interface, the internal fracture occurs easily in the undersurface of the chip closer to the cutting edge. Therefore, these positions become closer to the cutting edge as the feed rate increases. It should be noted that the cutting speed can also have a great impact on them as it can cause a surge in plastic deformation.

#### 3.1.7. In-Detail Evaluation of BUL’s Stability

**Figure 20:**
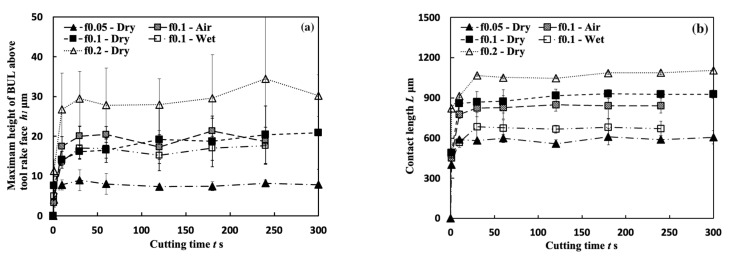
Variations in (**a**) height h1, and (**b**) contact length L of BUL with cutting time under different cutting conditions in cutting of Inconel 718(AG) at cutting speed 10 m/min, cutting depth 1 mm.

To actively and purposely utilize BUL in the dry cutting of Inconel 718(AG), it is also necessary to investigate the stability of the BUL during cutting. For this reason, variations in the height and length of the BUL with cutting time were examined. Here, to reduce the effect of the cutting tool nose, the cutting depth (1 mm) was kept the same. Figure 20 shows the variations in height *h*_1_ and contact length *L* of the BUL with cutting time at a cutting speed of 10 m/min under different feed rates and different cutting atmospheres within 300 s. As shown, *h*_1_ and *L* increases quickly at the beginning of cutting (~1 s), and it has a sight increasing trend after cutting 10–30 s. After cutting for 60 s, *h*_1_ and *L* are nearly constant. These results indicate that a stable BUL without the overall fall-off phenomenon forms on the tool rake for all cutting conditions used. The high stability of the BUL is confirmed. Meanwhile, it can also be found that the fluctuation of *h*_1_ is larger than the fluctuation of *L* when the feed rate increases above 0.1 mm/rev, especially the fluctuation of *h*_1_ for the feed rate of 0.2 mm/rev, which is a bit large. This phenomenon can be explained by the differences in the temperature distribution, contact condition, and friction on the tool rake face along the cutting tool edge direction during cutting. The larger feed rate can not only increase the maximum height of the BUL but also cause the inconsistency of the BUL’s height along the cutting tool edge direction, as shown in Figure 16, resulting in a large fluctuation of *h*_1_.

**Figure 21:**
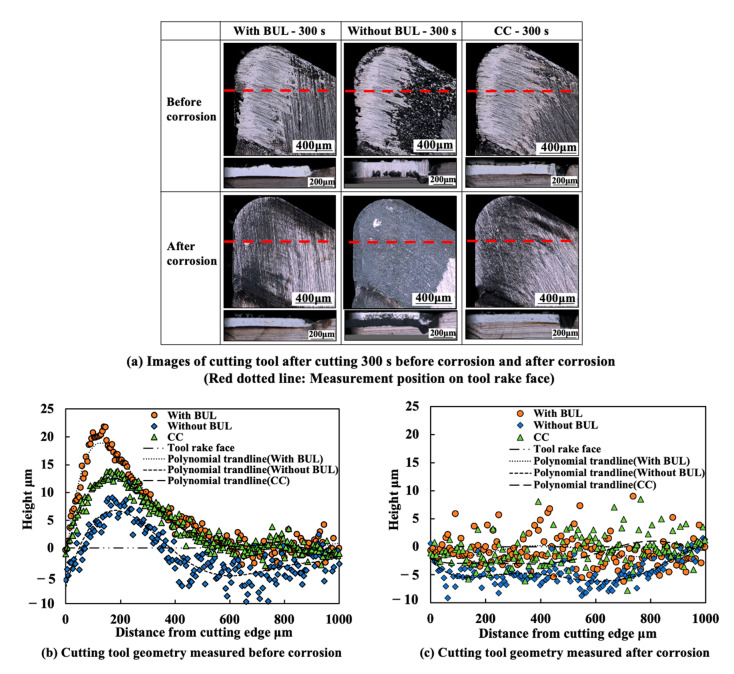
(**a**) Images of cutting tool after cutting 300 s before corrosion and after corrosion, and corresponding cutting tool geometries measured (**b**) before corrosion and (**c**) after corrosion (red dotted line in (**a**)) for three cutting tests in dry cutting of Inconel 718(AG) at cutting speed 10 m/min, feed rate 0.1 mm/rev, cutting depth 1 mm, cutting time 300 s.

Because the BUL strongly adheres to the tool rake face through the atomic-scale cluster sets [36], if the fragments of the BUL are forcefully ejected by the action of the chip or the workpiece, the tool material will be pulled away, and then tool wear or chipping appears. Using this feature, the stability of the BUL can also be evaluated by measuring the crater wear after a period of cutting time. For this reason, the used tools before and after corrosion were investigated. The microscope images and the corresponding cutting tool geometries of the cutting tools with and without the BUL after cutting for 300 s before and after corrosion are shown in Figure 21. Here, a cutting speed of 10 m/min, a feed rate of 0.1 mm/rev, a cutting depth of 1 mm, and dry cutting were selected. For comparison, the results of continuous cutting (cutting test “CC”) are also presented. As shown, it can be found that removing the BUL formed on the tool before each cut (cutting test “Without BUL”) can aggravate the crater wear even though the BUL reappears on the tool during cutting. However, leaving the BUL on the tool and using it for cutting (cutting test “With BUL”) can significantly prevent the occurrence of crater wear. This result confirms that the BUL can work as a protective layer and it is very stable without the overall fall-off phenomenon. The protective effect of BULs on the tool rake face will be discussed in detail in Section 3.3.

Additionally, it is worth stating that, as shown in Figure 21, although the crater wear eventually occurs on the tool rake face due to its more severe contact condition during continuous cutting, the depth of crater wear for cutting test “CC” is significantly smaller than that for the cutting test “Without BUL”. This result confirms that the stable BUL can also work as a protective layer during continuous cutting.

### 3.2. Influence of BUL on Cutting Process

Since the BUL is formed by the accumulation of workpiece material on the tool rake face and is very stable, it can change the tool shape and have a significant influence on the cutting processes. In order to actively and purposely utilize BUL during cutting, it is necessary and important to evaluate its influences on the cutting process. In this section, the influences of the BUL on cutting forces and surface roughness are investigated in detail.

#### 3.2.1. Cutting Forces

**Figure 22:**
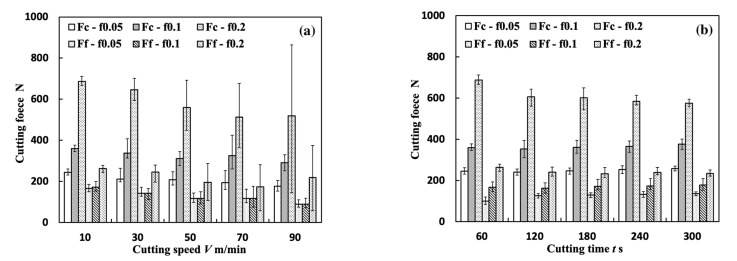
Variations in main cutting force $F_{c}$ and feed force $F_{f}$ (**a**) with cutting speed after cutting 60 s, and (**b**) with cutting time in dry cutting of Inconel 718(AG) at cutting speed 10 m/min with the feed rates 0.05, 0.1, and 0.2 mm/rev, cutting depth 1 mm.

Measuring the cutting forces during cutting allows the investigation of the stability of BULs and the influences of a BUL’s shape on the contact condition at the tool and chip (or workpiece) interface. The results of the main cutting force $F_{c}$ and feed force $F_{f}$ (average value during the steady cutting stage) in the dry cutting of Inconel 718(AG) at a cutting speed range of 10–90 m/min, with three different feed rates of 0.05, 0.1, and 0.2 mm/rev, a cutting depth of 1 mm, and a cutting time of 60 s are presented in Figure 22a. Considering the reported traditional evolution cycle time of BUE (10~200 Hz) [50], the measured cutting forces were filtered with a low-pass filter at 300 Hz. The maximum and minimum cutting forces are plotted as error bars. As shown, $F_{c}$ and $F_{f}$ show a decreasing trend as the cutting speed increases or the feed rate decreases. This can be explained as a result of the increase in the ductility of workpieces at high cutting speeds, which causes the high cutting temperature. And the larger feed rate contributes to a larger uncut chip thickness and a larger contact area, resulting in a larger cutting force. It can also be found that the amplitude variations in $F_{c}$ and $F_{f}$ have the minimum value at 10 m/min and increase as the cutting speed increases. This indicates that the BUL formation has few influences on the amplitude variations in cutting forces.

To study the stability of the BUL, variations in $F_{c}$ and $F_{f}$ with cutting time were also measured. Figure 22b shows the variations in $F_{c}$ and $F_{f}$ with cutting time in the dry cutting of Inconel 718(AG) at 10 m/min with three different feed rates of 0.05, 0.1, and 0.2 mm/rev and a cutting depth of 1 mm. As shown, $F_{c}$ and $F_{f}$ have only a slightly increasing trend with cutting time, except for the results of the feed rate of 0.2 m/rev. The amplitude variations in $F_{c}$ and $F_{f}$ are still small even after cutting for 300 s. This result confirms that the stability of the BUL is very high, and the BUL formation has few influences on the amplitude variations in cutting forces even after a long time of cutting.

**Figure 23:**
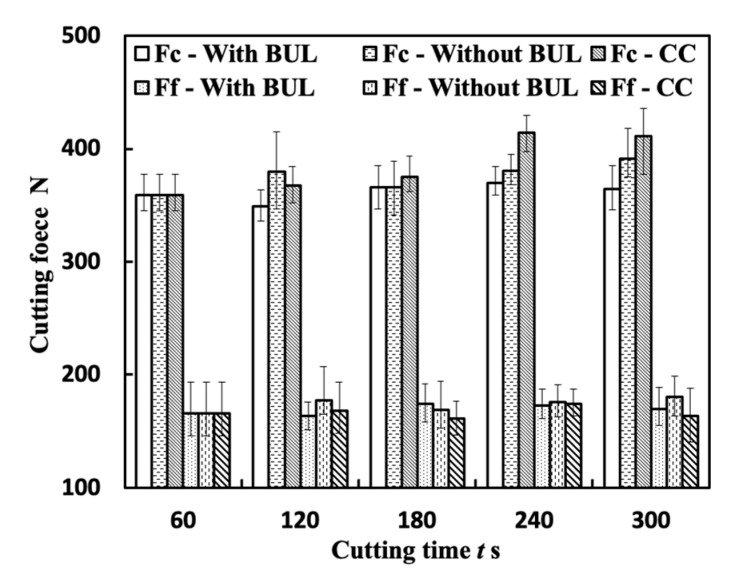
Variations in main cutting force $F_{c}$ and feed force $F_{f}$ with cutting time for three different cutting tests at cutting speed 10 m/min, feed rate 0.1 mm/rev, cutting depth 1 mm.

**Figure 24:**
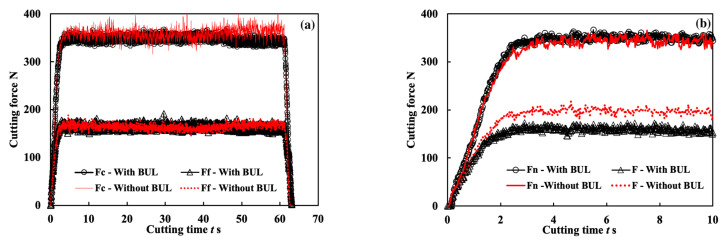
Variations in (**a**) measured main cutting force $F_{c}$ and feed force $F_{f}$, and (**b**) corresponding calculated normal force $F_{n}$ and frictional force *F* acted on the tool rake face with cutting time for cutting tests “with BUL” and “without BUL” from 60 s to 120 s at cutting speed 10 m/min, feed rate 0.1 mm/rev, cutting depth 1 mm.

Figure 23 shows the variations in $F_{c}$ and $F_{f}$ with cutting time for the three cutting tests “With BUL”, “Without BUL”, and “CC” in the dry cutting of Inconel 718(AG) at 10 m/min with a feed rate of 0.1 mm/rev and a cutting depth of 1 mm. Here, the results of the cutting test “With BUL” are the same as the results of the feed rate of 0.1 mm/rev shown in Figure 22. As shown in Figure 23, it can be found that $F_{c}$ and $F_{f}$ increase along with the cutting time for all cutting tests, but $F_{c}$ for the cutting tests “Without BUL” and “CC” increase remarkably compared with the cutting test “With BUL”. These phenomena can be explained as a combined result of the tool flank wear formation and the impact of the BUL on the cutting forces. Since the linear relationship between the width of flank wear and cutting forces has been reported in many previous studies [51,52], in this study, only the impact of the BUL on the cutting forces is investigated in detail. To reduce the influence of tool wear, the cutting forces for the cutting tests “With BUL” and “Without BUL” from 60 s to 120 s were considered. Figure 24a shows the cutting forces measurement obtained from the cutting tests “With BUL” and “Without BUL”. These results illustrate that the BUL can decrease $F_{c}$, but it has few influences on $F_{f}$. Since the BUL can also be formed for the cutting test “without BUL” after cutting 10 s, the forces applied on the tool rake face at the beginning of cutting (0–10 s) were calculated (Figure 24b). Here, the normal force $F_{n}$ and frictional force F worked on the rake face were calculated by $F_{n}=F_{c}cos\alpha_{0}-F_{f}sin\alpha_{0},\;F=F_{c}sin\alpha_{0}-F_{f}cos\alpha_{0}$ with the real tool rake angle $\alpha_{0}$ considering the BUL formation. The results in Figure 24b clarify that F for the cutting test “with BUL” is about 20% smaller than that for the cutting test “Without BUL”, which indicates that the BUL can decrease the frictional force F at the tool and chip interface. From this result, it can be concluded that the BUL can contribute to a reduction in the frictional force F, resulting in a small main cutting force $F_{c}$. This is consistent with the experimental results in Figure 23.

It should be noted that tool wear increases with cutting time, which can affect the forces acting on the tool. However, since the tool flank wear before cutting is almost the same for the two cases and the cutting time is too short, the influence of tool wear at the beginning of cutting for the two cases can be neglected.

#### 3.2.2. Surface Roughness

**Figure 25:**
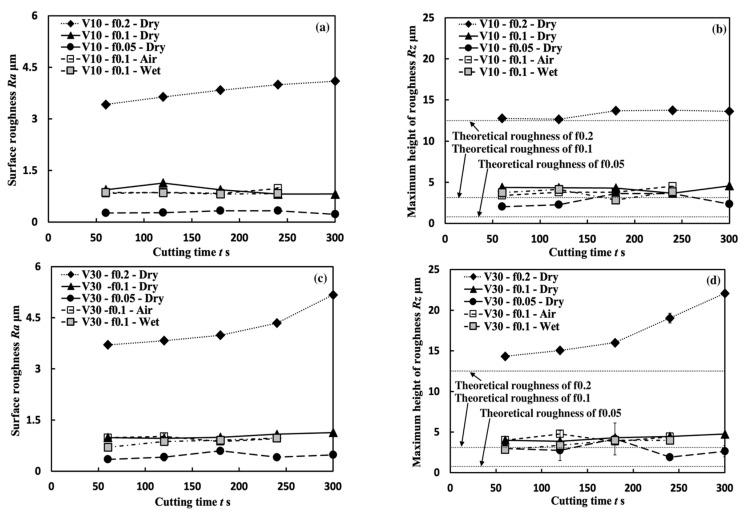
Variations in surface roughness *Ra* and *Rz* with cutting time in cutting of Inconel 718(AG): (**a**) *Ra*, and (**b**) *Rz* at cutting speed 10 m/min, and (**c**) *Ra*, and (**d**) *Rz* at 30 m/min with three feed rates 0.05, 0.1, and 0.2 mm/rev, three cutting atmospheres (dry, air, and wet), cutting depth 1 mm.

**Figure 26:**
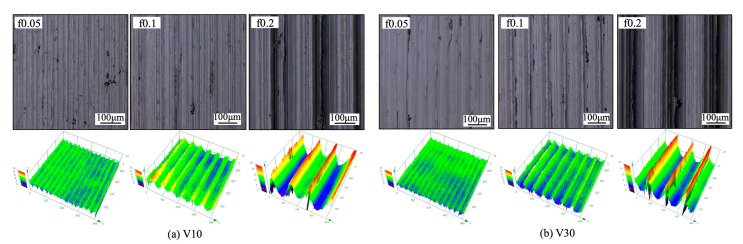
Images and three-dimensional geometries of machined surfaces after cutting 60 s in dry cutting of Inconel 718(AG) at cutting speeds (**a**) 10, and (**b**)30 m/min, three feed rates 0.05, 0.1, and 0.2 mm/rev, cutting depth 1 mm.

**Figure 27:**
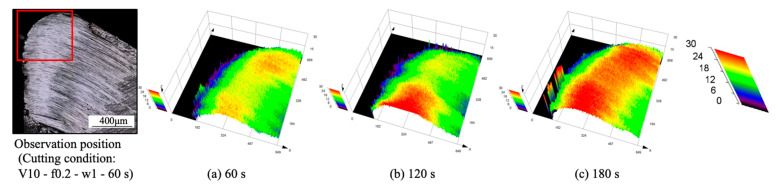
Three-dimensional geometries of tool nose after cutting (**a**) 60 s, (**b**) 120 s, and (**c**) 180 s in dry cutting of Inconel 718(AG) at cutting speed 10 m/min, feed rate 0.2 mm/rev, cutting depth 1 mm.

To evaluate the influence of the BUL, surface quality is investigated in terms of the evolution of surface roughness *Ra* and *Rz* with cutting time for each cutting test. Variations in surface roughness *Ra* and *Rz* along the feed direction with cutting time in the cutting of Inconel 718(AG) at cutting speeds of 10 and 30 m/min with three feed rates of 0.05, 0.1, and 0.2 mm/rev, three cutting atmospheres (dry, air, and wet), and a cutting depth of 1 mm are presented in Figure 25. For comparison, the theoretical roughness *Rz* for feed rates of 0.05, 0.1, and 0.2 mm/rev is also plotted. As shown, it can be observed that the larger feed rate leads to a larger *Ra* or *Rz*, and no significant variation in *Ra* and *Rz* can be observed in all cases. It can also be observed that neither *Ra* nor *Rz* increases significantly with the cutting time at feed rates of 0.05 and 0.1 mm/rev under different cutting atmospheres. However, as shown, at a feed rate of 0.2 mm/rev, there is a more significant increasing trend for *Ra* and *Rz* at 30 m/min than that at 10 m/min. To explain this phenomenon, the 3-D geometries of machined surfaces in the dry cutting of Inconel 718(AG) at 10 and 30 m/min were investigated (Figure 26). It can be seen that the width of the feed marks becomes larger and their depth becomes deeper as the feed rate increases up to 0.2 mm/rev. This result indicates that the tool nose’s shape can significantly affect the surface roughness at a feed rate of 0.2 mm/rev. Therefore, the slightly increasing trends of *Ra* and *Rz* at 10 m/min and a feed rate of 0.2 mm/rev can be explained by the over-growth of the BUL near the tool nose (Figure 27). Since the BUL does not fall off and can replace the tool to perform the actual cutting, *Ra* and *Rz* increase slightly as the BUL grows. In contrast, the significantly increasing trends of *Ra* and *Rz* at 30 m/min and a feed rate of 0.2 mm/rev can be explained by a rapid increase in the tool nose notch wear, which can reduce the tool nose radius.

Comparing the results under different cutting atmospheres at 10 m/min, it can be found that the average *Ra* from 60 s to 300 s for the dry atmosphere (*Ra*: 0.927 ± 0.133, *Rz*: 4.257 ± 0.339) is only about 5.7% larger than that for the air atmosphere (*Ra*: 0.876 ± 0.066, *Rz*: 3.868 ± 0.462), and about 10.6% larger than that for the wet atmosphere (*Ra***:** 0.838 ± 0.022, *Rz*: 3.641 ± 0.563), which indicates that using the BUL for dry cutting can still obtain better surface roughness. From these results, it can be concluded that the BUL does not lead to an increase in the surface roughness but can maintain the surface roughness and bring the surface roughness close to the theoretical value. However, it should be noted that if the BUL grows excessively near the tool nose, it will also slightly affect the surface roughness.

### 3.3. Protective Effect of BUL

In this section, the protective effect of the BUL, the protective effect of FBU, and the influence of the BUL’s height on its protective effect are investigated.

#### 3.3.1. Overall Evaluation of the Protective Effect of BUL in Dry Cutting of Inconel 718(AG)

To study the protective effect of the BUL, various tool wear tests were carried out. Firstly, a comparative experiment was used to investigate the protective effect of the BUL.

**Figure 28:**
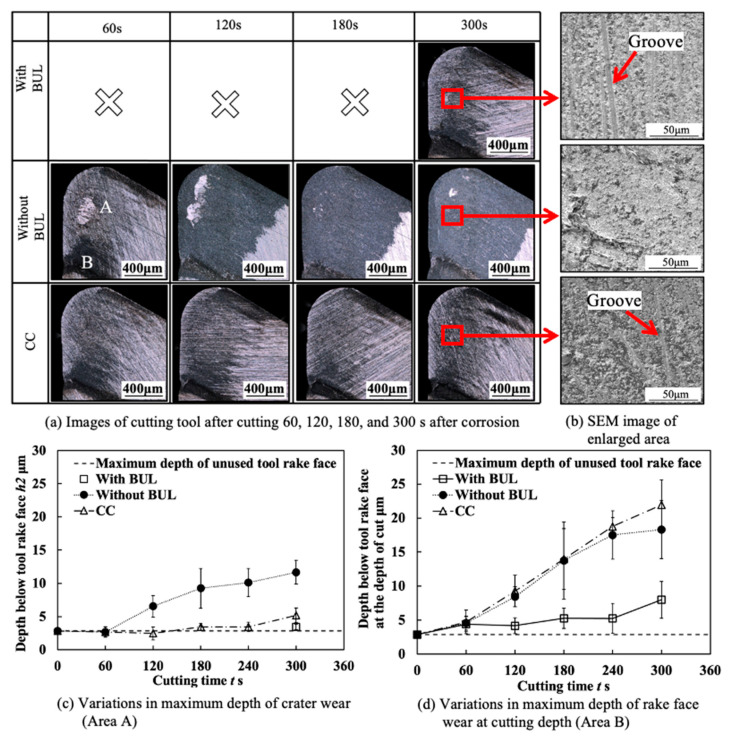
(**a**) Images of cutting tool after cutting 60, 120, 180, and 300 s after corrosion, (**b**) corresponding SEM images of enlarger area of tool rake face after cutting 300 s, and variations in (**c**) maximum depth of crater wear (Area A), and (**d**) maximum depth of rake face wear at cutting depth (Area B) with cutting time for three different cutting tests in dry cutting of Inconel 718(AG) at cutting speed 10 m/min, feed rate 0.1 mm/rev, cutting depth 1 mm.

**Figure 29:**
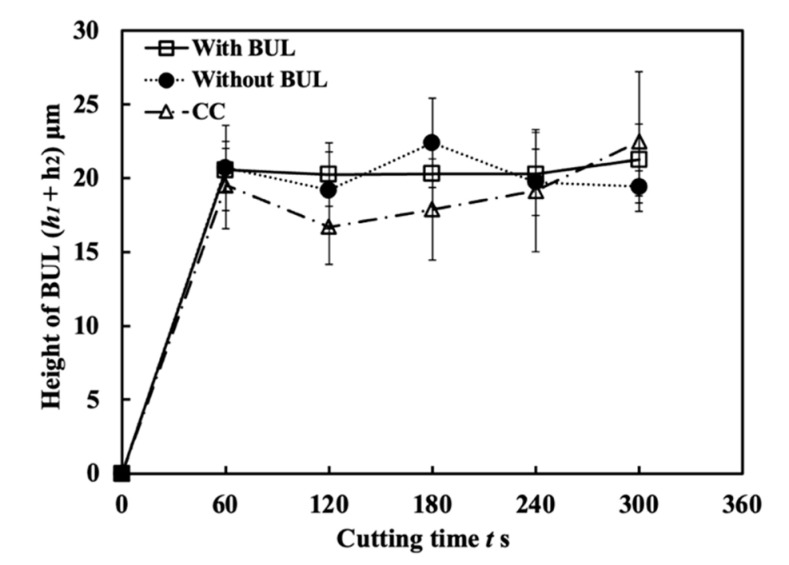
Variations in maximum height of BUL (*h*_1_ + *h*_2_) considering the crater wear with cutting time for different cutting tests in dry cutting of Inconel 718(AG) at cutting speed 10 m/min, feed rate 0.1 mm/rev, cutting depth 1 mm.

Figure 28 shows the microscope images of the cutting tool after corrosion and the variations in the depth of crater wear with cutting time for three different cutting tests in the dry cutting of Inconel 718(AG) at a cutting speed of 10 m/min, a feed rate of 0.1 mm/rev, and a cutting depth of 1 mm. Here, the mean maximum depth of the original grooves generated on the tool surface during fabrication (its mean maximum depth is 2.842±0.434 μm) is also presented. The results shown in Figure 28a,c illustrate that for the cutting test “Without BUL”, the significant crater wear appears on the main tool rake face (Area A) after cutting 120 s; for the cutting test “CC”, the slight crater wear occurs after cutting 180 s; and for the cutting test “With BUL”, there is almost no crater wear after cutting 300 s. And the depth of crater wear is increasing with cutting time for the cutting tests “Without BUL” and “CC”. From the detailed observation of the tool rake face shown in Figure 28b, the obvious original grooves remain on the tool rake face after cutting for 300 s for the cutting tests “With BUL” and “CC”. In contrast, for the cutting test “Without BUL”, the delamination of tool material occurs, and the cemented carbide particles are exposed on the worn tool rake face. These results confirm that the BUL has a protective effect and can significantly protect the tool rake face from crater wear. The crater wear formation after cutting for 180 s for the cutting test “CC” can be attributed to the decrease in the height of the BUL due to its more severe contact condition during continuous cutting. As shown in Figure 29, it can be clearly seen that the total height of the BUL (*h*_1_ + *h*_2_) for the cutting test “CC” becomes lower than that for the cutting test “With BUL” after cutting for 120 s.

**Figure 30:**
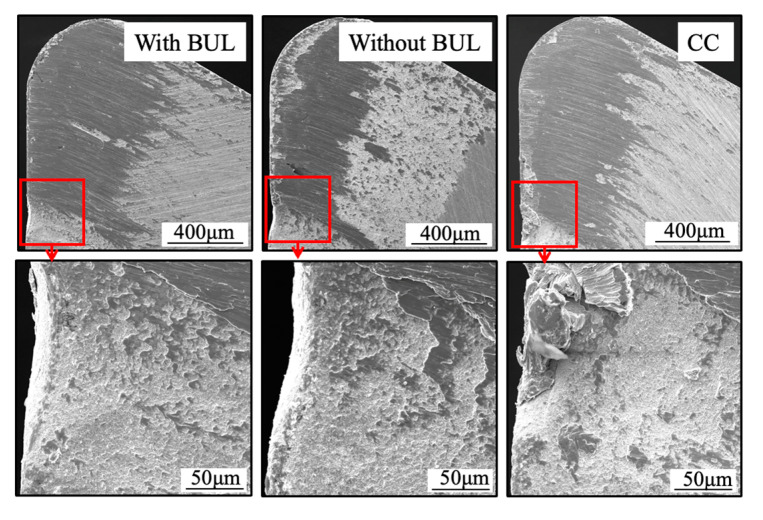
SEM images of tool rake face before corrosion for three different cutting tests in dry cutting of Inconel 718(AG) at 10 m/min (feed rate 0.1 mm/rev, cutting depth 1 mm, cutting time 300 s).

As shown in Figure 28a, it can also be found that there is significant wear on the tool rake face at the cutting depth (Area B) in all cases. For this reason, the depth of rake face wear at the cutting depth was also measured (Figure 28d), and the detailed observations on the tool rake face after cutting for 300 s before corrosion were also applied (Figure 30). It can be seen in Figure 28d that the depth of rake face wear at the cutting depth is increasing with the cutting time in all cases. However, the depth of rake face wear at the cutting depth after cutting 300 s for the cutting test “With BUL” is about 2.5 times smaller than that for the cutting test “Without BUL” or “CC”. This phenomenon can be explained as a result of the smooth and relatively stable contact between the work-hardened chip and tool rake face at the cutting depth, which increases the mechanical wear of tool. As shown in Figure 30, the rake face wear at the cutting depth shows a narrow and long sharp shape, and the cemented carbide particles are exposed without the BUL formation. However, since the BUL can separate the tool from the chip at the cutting depth to a certain extent after formation, the BUL can inhibit the formation of rake face wear at the cutting depth to a certain extent. The protective effect of the BUL on the tool rake face was verified.

**Figure 31:**
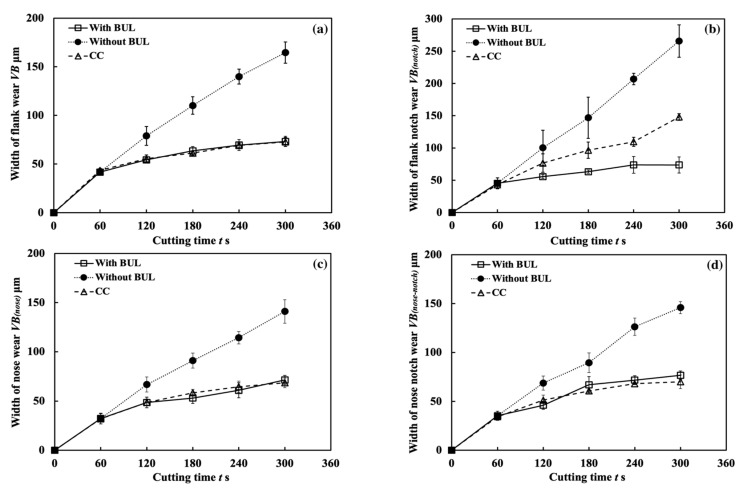
Variations in (**a**) flank wear *VB*, (**b**) flank notch wear *VB_(notch)_*, (**c**) nose wear *VB _(nose)_*, and (**d**) nose notch wear *VB_(nose-notch)_* with cutting time for three different cutting tests in the dry cutting of Inconel 718(AG) at cutting speed 10 m/min, feed rate 0.1 mm/rev, cutting depth 1 mm.

**Figure 32:**
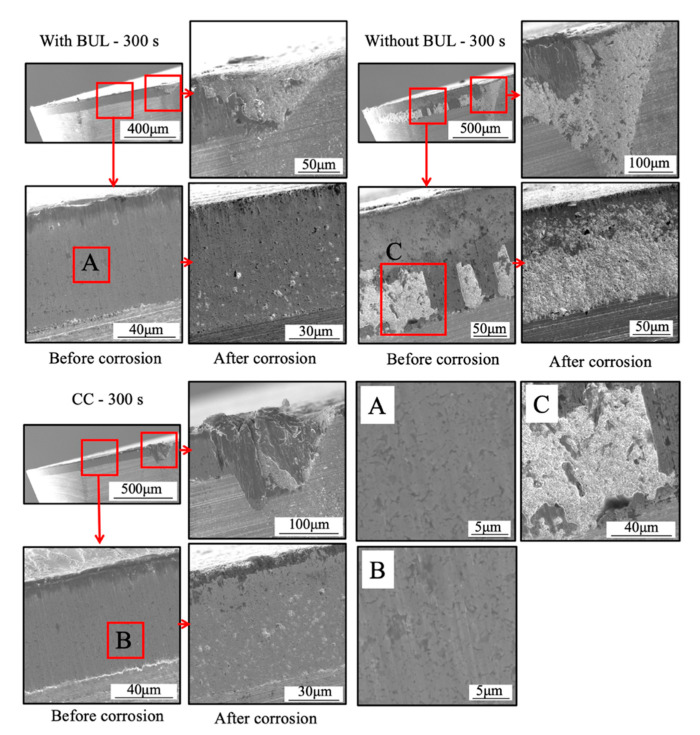
SEM images of tool flank face before and after corrosion for three different cutting tests in dry cutting of Inconel 718(AG) at cutting speed 10 m/min, feed rate 0.1 mm/rev, cutting depth 1 mm, cutting time 300 s.

Figure 31 shows the variations in the tool flank wear *VB*, flank notch wear *VB_(notch)_*, nose wear *VB_(nose)_*, and nose notch wear *VB_(nose-notch)_* with cutting time for three different cutting tests in the dry cutting of Inconel 718(AG) at a cutting speed of 10 m/min, a feed rate of 0.1 mm/rev, and a cutting depth of 1 mm. As shown, both tool flank wear and nose wear are increasing with cutting time in all cases. However, the tool flank wear and nose wear for the cutting test “With BUL” are lower than those for the cutting test “without BUL” from 120 s, and the difference between them increases with cutting time. Meanwhile, *VB*, *VB_(nose)_*, and *VB_(nose-notch)_* for the cutting test “CC” are similar to those for the cutting test “With BUL”, but *VB_(notch)_* for the cutting test “CC” is larger from 120 s. These phenomena can be explained as a result of the protective effects of BUL and FBU formed on the worn tool flank face. Since the size of the BUL (Figure 29) and the cutting conditions (cutting speed, etc.) are almost the same for the three different cutting tests, the protective effects of the BUL on the tool flank face and nose face are similar. Therefore, the protective effect of the FBU may play an important role in these phenomena. For illustration, the tools after cutting 300 s before and after corrosion were investigated. As shown in Figure 32, only in the cutting tests “With BUL” and “CC” can FBU completely cover the worn tool flank face, and the worn surface is relatively smooth without apparent scratching grooves and exposed carbide particles. These results indicate that FBU can prevent the tool from further wear to a certain extent. On the other hand, it can also be found that the larger FBU (or adhesion) can form on the tool flank face at the cutting depth for the cutting test “CC”. Due to the lower stability of the larger FBU, the flank notch wear for the cutting test “CC” increases quickly compared with that for the cutting test “With BUL”, as shown in Figure 31b.

#### 3.3.2. Protective Effect of FBU on Tool Flank Face

As mentioned above, it was confirmed that FBU may also prevent the tool flank face from further wear to a certain extent. To distinguish the protective effects of the BUL and FBU on the tool flank face, the FBU formation process and its protective effect on the tool flank face under different cutting conditions are investigated in this section.

**Figure 33:**
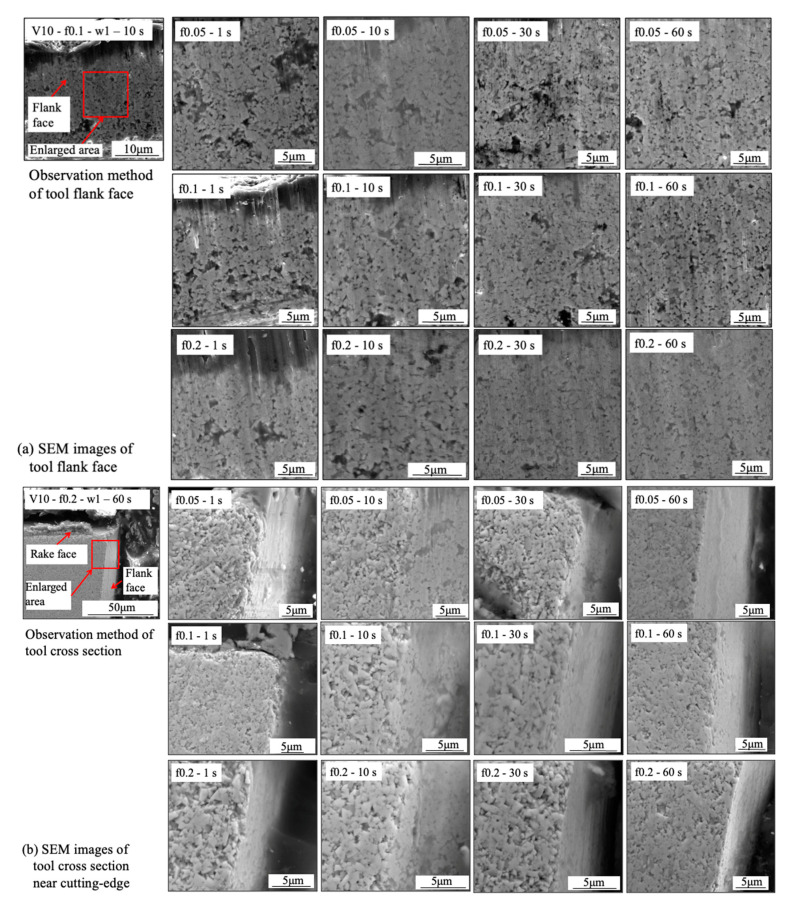
SEM images of (**a**) tool flank face and (**b**) tool cross section near cutting-edge in dry cutting of Inconel 718(AG) at cutting speed 10 m/min, feed rates 0.05, 0.1, and 0.2 mm/rev, cutting depth 1 mm, cutting time 1, 10, 30, 60 s.

**Figure 34:**
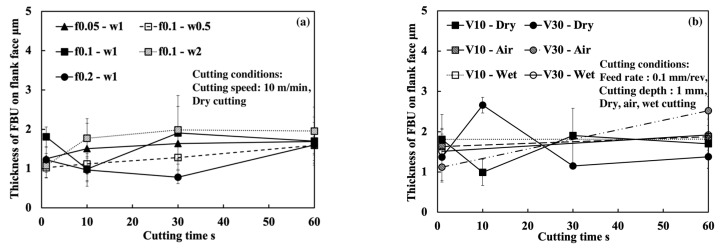
Variations in maximum thickness of FBU on the flank face with cutting time in cutting of Inconel 718(AG): (**a**) at cutting speed 10 m/min, feed rates 0.05, 0.1, and 0.2 mm/rev, cutting depths 0.5, 1, and 2 mm, dry cutting atmosphere, (**b**) at cutting speeds 10 and 30 m/min, feed rate 0.1 mm/rev, cutting depth 1 mm, dry, air and wet atmospheres.

Firstly, the FBU formation process is investigated. For illustration, SEM images of the tool flank face and the tool cross section near the cutting edge for four different cutting times (1, 10, 30, and 60 s) in the dry cutting of Inconel 718(AG) at a cutting speed of 10 m/min, feed rates of 0.05, 0.1, and 0.2 m/min, and a cutting depth of 1 mm are shown in Figure 33. It can be verified that FBU can form on the entire worn flank face quickly after cutting (~1 s), but it remains very thin even after cutting for 60 s since the shape of WC grain can also be observed on the worn flank face. In addition, as shown in Figure 33b, the thickest FBU occurs near the cutting edge, and its thickness decreases with the distance from the cutting edge. Therefore, to evaluate the thickness of FBU, the maximum thickness of FBU near the cutting edge was measured in this study (Figure 34). Here, for comparison, the results at 30 m/min were also measured. The results shown in Figure 34 illustrate that FBU is very thin (around 1–2 μm) and its thickness does not change too much over time in all cases, which indicates that the cutting conditions have few or no influences on the FBU formation. This FBU formation process has also been proven by the EPMA analysis. From the EPMA analysis results in our previous study [15], it can be confirmed that FBU is not an accumulation of tool wear debris but an accumulation of the workpiece material, and it covers the entire worn flank face after cutting ~1 s, even if it is very thin.

On the other hand, it can also be seen in Figure 33 that the worn flank face is very smooth without obvious scratching grooves and exposed carbide particles in all cases. These results indicate that the thin FBU can separate the tool flank face from the workpiece and decrease adhesive wear, abrasive wear, etc., resulting in smaller wear. Therefore, it can be concluded that FBU can work as a protective layer and protect the flank face from further wear. However, since the thickness of FBU is almost the same (Figure 34), it can be said that the protective effect of FBU is almost similar under different cutting conditions. Therefore, it can be further concluded that the flank wear progression under different cutting conditions can be used to investigate the protective effect of the BUL on the tool flank face in the dry cutting of Inconel 718(AG). However, it should be noted that, as shown in Figure 32, the coverage area of FBU on the worn tool flank face can greatly affect its protective effect. Therefore, in this study, only the cutting test “With BUL” is considered.

As shown in Figure 33a, many pits can be observed on the relatively smooth worn flank face in all cases. The pit formation can be related to the removal of the Co binder phase from the grain boundaries or the pull-out of unsupported WC grains due to the smooth worn flank face. Meanwhile, as shown in Figure 33b, no fracture of WC grains in the direction perpendicular to the worn flank wear can be observed. These results indicate that the fall-off of WC grains and the fatigue microcracks in the sliding direction of workpiece are the main forms of wear formation. Based on these results, it can be concluded that the adhesive-induced wear (adhesive wear and high-cycle fatigue wear) [12], rather than abrasive wear, is the major wear mechanism of flank wear with the FBU formation.

#### 3.3.3. Protective Effect of BUL on Tool Flank Face

In this section, the protective effect of the BUL and the influence of the BUL’s height on its protective effect are investigated. Based on the results above, five cutting speeds (10, 30, 50, 70, and 90 m/min), three feed rates (0.05, 0.1, and 0.2 mm/rev), and the same cutting depth of 1 mm were selected for the cutting tests. The total cutting time was set to 300 s.

**Figure 35:**
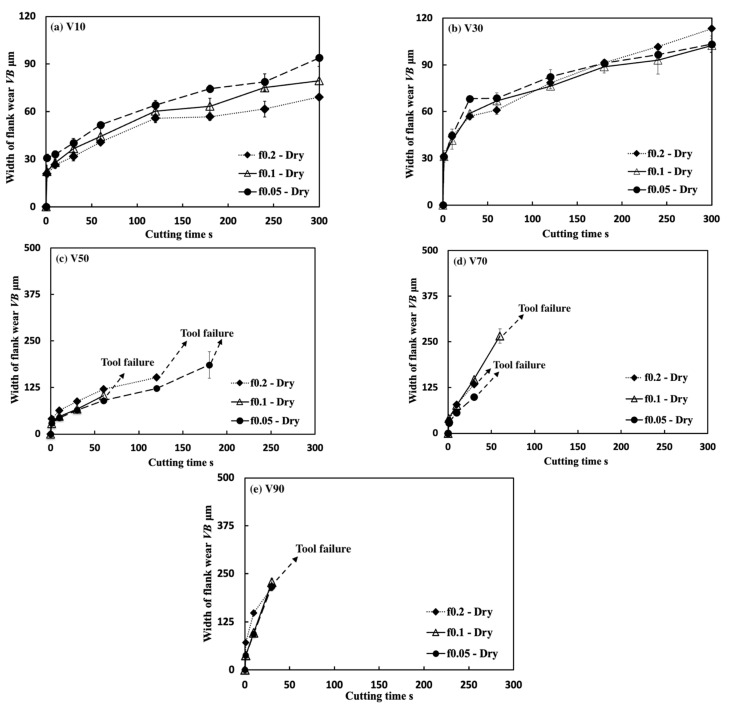
Variations in flank wear *VB* with cutting time in dry cutting of Inconel 718(AG) at cutting speeds (**a**) 10, (**b**) 30, (**c**) 50, (**d**) 70, and (**e**) 90 m/min, feed rates 0.05, 0.1, and 0.2 mm/rev, cutting depth 1 mm.

**Figure 36:**
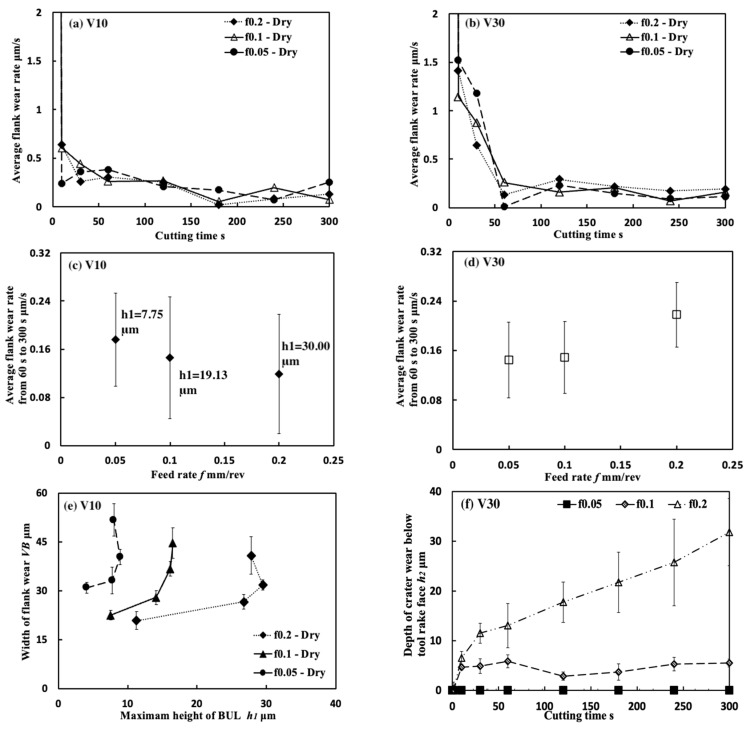
Variations in average flank rate with cutting time at cutting speeds (**a**) 10, and (**b**) 30 m/min, variations in average flank wear rate from 60 s to 300 s with feed rate at cutting speeds (**c**) 10, and (**d**) 30 m/min, (**e**) relation between *VB* and height of BUL *h_1_* from 1 s to 60 s at 10 m/min, and (**f**) variations in crater wear below tool rake face $h_{2}$ with cutting time at 30 m/min in dry cutting of Inconel 718(AG)) with three feed rates 0.05, 0.1, and 0.2 mm/rev, cutting depth 1 mm.

Figure 35 shows the variations in *VB* with cutting time under different cutting conditions. As shown, it can be found that the tool can only exhibit a long tool life in the low cutting speed range of 10–30 m/min in all cases. This result confirms that Inconel 718(AG) is difficult to cut at a cutting speed above 50 m/min. Meanwhile, it can be found that at 10 m/min, *VB* after cutting the same time decreases as the feed rate increases, and *VB* after cutting for 300 s at the feed rate of 0.2 mm/rev is about 13% smaller than that at 0.1 mm/rev and about 26% smaller than that at 0.05 mm/rev. It can be also found that at 30 m/min, although the progression of flank wear at different feed rates exhibits an almost similar pattern, *VB* after cutting for 300 s at the feed rate of 0.1 mm/rev is still slightly smaller than that at 0.05 mm/rev, and *VB* after cutting for 300 s at 0.2 mm/rev is larger than that at 0.05 or 0.1 mm/rev. These phenomena can be explained as the result of the protective effect of the BUL, which can not only greatly reduce the average tool flank wear rate but also protect the tool from wear at the beginning of cutting. To validate this explanation, the variations in average flank rate with cutting times at 10 and 30 m/min, the variations in average flank wear rate from 60 s to 300 s with feed rates at 10 and 30 m/min, and the relation between *VB* and the height of BUL *h*_1_ from 1 s to 60 s at 10 m/min were investigated (Figure 36). As shown in Figure 36a, it can be found that at 10 m/min, the average tool flank wear rate becomes low rapidly after cutting 1 s, and the changing trend of the average tool flank wear rate between the tool’s initial wear stage (~60 s) and the steady-state wear stage (>60 s) is relatively flat. Meanwhile, it can be found in Figure 36c,e that both *VB* in the tool initial wear stage (~60 s) and the average tool flank wear rate in the steady-state stage (>60 s) correspondingly decrease as the feed rate or *h_1_* increases, which confirms that the BUL has the protective effect and its height *h_1_* can play a great role in its protective effect. This result is also in close agreement with the findings in the dry cutting of stainless steel SUS304 [13].

On the other hand, as shown in Figure 36b,d, it can be found that at 30 m/min, although *VB* in the tool initial wear stage (~60 s) at different feed rates is very close, the average flank wear rate in the steady-state stage (>60 s) has an increasing trend as the feed rate increases up to 0.2 mm/rev due to the severe cutting condition at larger feed rates. Considering the BUL formation condition (Figure 15), it can be said that the BULs formed at 30 m/min can only maintain a certain level of their protective effect, and only reduce the average tool flank wear rate in the steady-state wear stage (>60 s). Similar results can also be obtained on the tool rake face for crater wear (Figure 36f). It can be seen in Figure 36f that at 30 m/min, only the BUL formed when the feed rate is 0.1 mm/rev or less can prevent the rake face from further crater wear.

From these results, it can thus be concluded that the BUL can work as a protective layer, which can not only prevent tool crater wear but also reduce tool flank wear, and its height can play a great role in its protective effect. However, it should be noted that FBU also has a protective effect on the tool flank face, but because it is very thin, it does not play a major role in the above phenomena.

Based on the results in Section 3, it can be concluded that we can realize the SPT in dry cutting of Inconel 718(AG) by actively and purposely utilizing BUL in the cutting speed range below 30 m/min, especially at 10 m/min, at which lower tool wear formation, longer tool life, and better surface roughness can be obtained. In the next section, we will give a detailed discussion on the effectiveness and generalizability of the SPT method.

## 4. Validation Experiments of Proposed SPT Method

### 4.1. Comparison with the Results under Air and Wet Cutting Atmospheres

**Figure 37:**
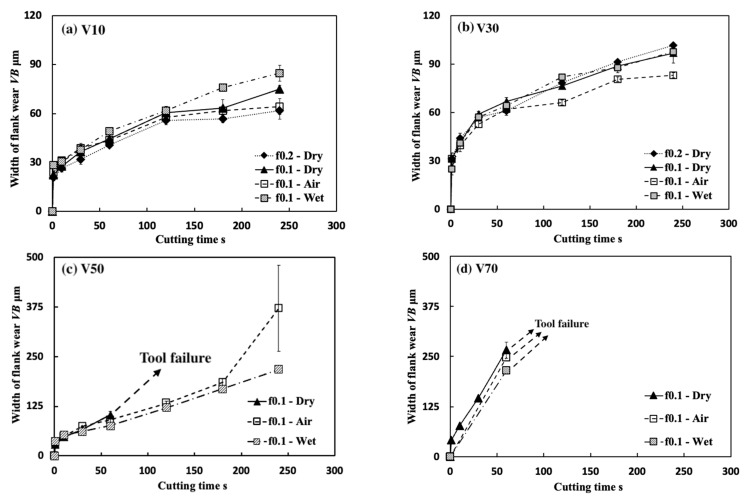
Variations in flank wear *VB* with cutting time in dry, air, and wet cutting of Inconel 718(AG) at cutting speeds (**a**) 10, (**b**) 30, (**c**) 50, and (**d**) 70 m/min, feed rate 0.1 mm/rev, cutting depth 1 mm.

Figure 37 shows the variations in *VB* with cutting time in air and wet cutting of Inconel 718(AG) at different cutting speeds of 10–70 m/min with a feed rate of 0.1 mm/rev and a cutting depth of 1 mm. The total cutting time was set to 240 s. For comparison, the results of dry cutting are also presented. As shown, at 10 m/min, *VB* after cutting for 240 s for the dry cutting atmosphere at the feed rate of 0.1 mm/rev is about 11.3% smaller than that for the wet cutting atmosphere, and about 16.9% larger than that for the air cutting atmosphere, but *VB* for the dry cutting atmosphere at the feed rate of 0.2 mm/rev is about 4.1% smaller than that for the air cutting atmosphere at 0.1 mm/rev. At 30 m/min, *VB* after cutting for 240 s for the dry cutting atmosphere at the feed rate of 0.1 mm/rev is about 11.7% larger than that for the air cutting atmosphere, but it is still about 4.9% smaller than that for the wet cutting atmosphere. These results confirmed that we can substantially reduce tool wear and extend tool life by utilizing the appropriate size of the BUL in the dry cutting of Inconel 718(AG) without the use of a cutting fluid system. Meanwhile, it can be found that at the same feed rate, *VB* for the air cutting atmosphere at 10 and 30 m/min is smaller than that for the dry cutting atmosphere. This can be related to the fact that a higher BUL can be formed on the tool in the air cutting atmosphere compared with the dry cutting atmosphere (Figure 20), which also indicates that the air cutting atmosphere may enhance the protective effect of the BUL on the flank face. On the other hand, the results in Figure 37 also illustrate that although the air and wet cutting atmospheres can increase tool lifespan at cutting speeds above 50 m/min compared with the dry cutting atmosphere, Inconel 718(AG) is still difficult to cut at cutting speeds above 50 m/min under the air or wet atmosphere.

**Figure 38:**
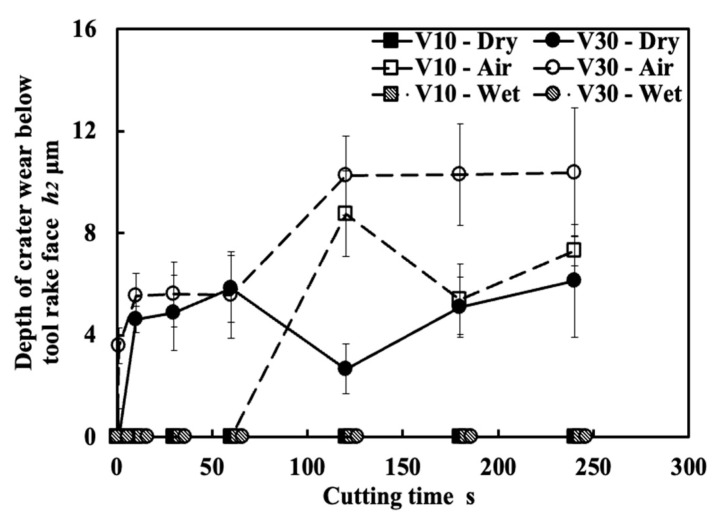
Variations in depth of crater wear $h_{2}$ with cutting time in dry, air, and wet cutting of Inconel 718(AG) at cutting speeds 10 and 30 m/min, feed rate 0.1 mm/rev, cutting depth 1 mm.

Figure 38 shows the corresponding variations in the depth of crater wear *h*_2_ with cutting times at 10 and 30 m/min in the dry, air, and wet cutting of Inconel 718(AG) with a feed rate of 0.1 mm/rev and a cutting depth of 1 mm. As shown, it can be found that at 10 m/min, crater wear begins to occur from 120 s only for the air cutting atmosphere, and at 30 m/min, it occurs at the beginning of cutting (~10 s) for the dry and air cutting atmospheres. However, in all cases, the increasing trend of *h*_2_ decreases after cutting 180 s. And there is no crater wear in wet cutting at 10 and 30 m/min and in dry cutting at 10 m/min. These results indicate that the BUL can function as a protective layer under different cutting atmospheres.

From these results, it can be concluded that the effectiveness of the proposed SPT method was verified. It can also be concluded that by utilizing the appropriate size of the BUL, the cutting tool used in the dry cutting atmosphere can also obtain a lower tool wear than that used in the air or wet cutting atmosphere.

### 4.2. Comparison with the Results when dry Cutting Inconel 718(ST)

**Figure 39:**
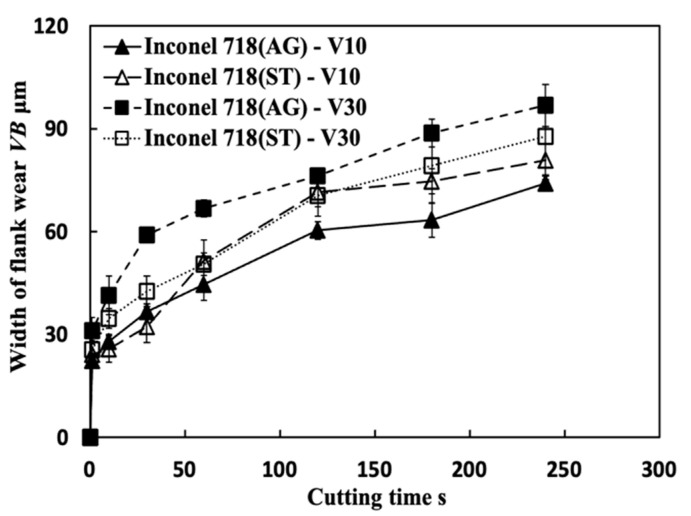
Variations in flank wear *VB* with cutting time in dry cutting of Inconel 718(ST) at cutting speeds 10, and 30 m/min, feed rate 0.1 mm/rev, cutting depth 1 mm.

**Figure 40:**
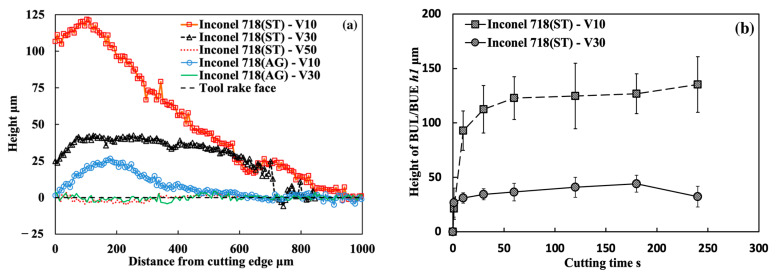
(**a**) BUL/BUE’s profiles after cutting 60 s at cutting speed 10, 30 and 50 m/min, and (**b**) variations in height of BUL/BUE with cutting time at cutting speed 10, and 30 m/min in dry cutting of Inconel 718(ST) with feed rate 0.1 mm/rev, cutting depth 1 mm.

Figure 39 shows the variations in *VB* with cutting time in the dry cutting of Inconel 718 (ST) at cutting speeds of 10 and 30 m/min, a feed rate of 0.1 mm/rev, and a cutting depth of 1 mm. The total cutting time was set to 240 s. For comparison, the results of dry cutting Inconel 718(AG) with the same cutting conditions are also presented. As shown, at 10 m/min, *VB* after cutting 240 s for Inconel 718(AG) is about 7.1% smaller than that for Inconel 718(ST), but at 30 m/min, *VB* after cutting 240 s for Inconel 718(AG) is about 5.8% larger than that for Inconel 718(ST). In general, there is a positive correlation between the hardness of the workpiece and flank wear [53]. Therefore, the smaller *VB* at 10 m/min for Inconel 718(AG), which is harder than Inconel 718(ST) (Table 2), can be related to the protective effect of the BUL. Another possible reason for this phenomenon can be related to the BUE formation at 10 m/min in the dry cutting of Inconel 718(ST). Figure 40a shows the adhesions’ profiles in the dry cutting of Inconel 718(ST) at 10, 30, and 50 m/min, a feed rate of 0.1 mm/rev, and a cutting depth of 1 mm. For comparison, the results of dry cutting Inconel 718(AG) are also presented. It can be found in Figure 40a that the height of adhesion formed at 10 m/min in the dry cutting of Inconel 718(ST) is above 100 μm and it can grow up to a noticeable size around the cutting edge. From the results of surface roughness at 10 m/min, it can also be found that, compared with *Rz* for Inconel 718(AG) (*Ra*: 0.783±0.022 μm, *Rz*: 3.443±0.130 μm), *Rz* for Inconel 718(ST) (*Ra*: 0.993±0.028 μm, *Rz*: 4.923±0.223 μm) is about 57% larger than the theoretical surface roughness *Rz* = 3.13 μm with a feed rate of 0.1 mm/rev, which indicates that the adhesion formed at 10 m/min in the dry cutting of Inconel 718(ST) can lead to a more severe over-cutting phenomenon, resulting in the reduction in surface roughness. The variations in the height of adhesion with cutting time in the dry cutting of Inconel 718(ST) were also measured. It can be found in Figure 40b that the amplitude variation in the height of adhesion formed at 10 m/min is very large, which indicates that the adhesion is not stable. These results confirm that the adhesion formed at 10 m/min in the dry cutting of Inconel 718(ST) should be called BUE, which is unstable and can cause the tool wear when its fragments are carried away by the chip or workpiece. On the other hand, in general, a higher cutting speed leads to a higher cutting temperature and increases wear formation. However, as shown in Figure 39, *VB* for Inconel 718(ST) exhibits an almost similar progression at 10 and 30 m/min, while *VB* for Inconel 718(AG) at 30 m/min is about 21 μm larger than that at 10 m/min from 30 s. This can be explained as a result of the protective effect of the BUL in the dry cutting of Inconel 718(ST) at 30 m/min since a stable BUL can be formed (Figure 40). In addition, the BUL/BUE formation condition in the dry cutting of Inconel 718(ST) with a feed rate of 0.1 mm/rev and a cutting depth of 1 mm can be given below: BUE occurs at a low cutting speed (about 10 m/min); the BUL can be formed on the rake face in the cutting speed range of 30–50 m/min.

From these results, it can be concluded that the material microstructure and mechanical properties of Inconel 718 have major effects on the BUL/BUE formation, and the SPT can also be realized even in dry cutting of Inconel 718(ST). It can also be concluded that the BUL formation cutting speed range can be increased by modifying the material microstructure of Inconel 718, finally increasing the cutting speed range of SPT that can be effectively used for dry cutting Inconel 718. The generalizability of the proposed SPT method was verified. Furthermore, in the same way as the development of free-machining steel with improved machinability and chip control of carbon steel, we can expect the development of a free-machining Inconel 718 by actively and purposely utilizing BULs.

## 5. Conclusions

In this study, a novel approach for dry cutting Inconel 718 in a more sustainable and low-cost way by actively and purposely utilizing the built-up layer (BUL), which can be called the self-protective tool (SPT) method, was proposed and investigated in detail. Various cutting experiments were conducted using the age-treated Inconel 718 and relatively inexpensive uncoated cemented carbide tools to investigate the BUL formation phenomenon and the effectiveness of the SPT method. The following conclusions can be drawn:

(1) Only BUL, not BUE, is formed on the tool rake face when cutting the age-treated Inconel 718. By modifying the material microstructure of Inconel 718, we can control the BUL and BUE formation cutting speed range and the height of BUL.
(2) The cutting speed plays a decisive role in the BUL formation condition. In most cases, the BUL only occurs in the cutting speed range of ~30 m/min, and this cutting speed range can increase to ~50 m/min or decrease to ~10 m/min depending on the cutting condition. The feed rate has a more significant influence on the BUL formation cutting speed range compared with the cutting depth and cutting atmosphere. And the cutting-edge radius has few or no influences on the BUL formation.
(3) The height and contact length of the BUL decrease as the cutting speed increases, and increase as the feed rate or cutting depth increases. A larger cutting-edge radius can reduce the height of the BUL without affecting its length. The air cutting atmosphere tends to increase the height and length of the BUL, while the wet cutting atmosphere tends to decrease them. These relationships between the size of the BUL and cutting conditions can be used to develop an effective BUL control system.
(4) The stability of the BUL is very high, and the BUL can not only significantly protect the tool from wear but also reduce friction at the tool–chip interface and maintain surface roughness. The height of the BUL can play a very important role in its protective effect, and a higher BUL can improve its protective effect. This indicates that we can realize the SPT when dry cutting Inconel 718 by actively and purposely utilizing the BUL and control the SPT by controlling the height of the BUL.
(5) Not only the BUL but also FBU has a protective effect on the tool, and the adhesive-induced wear may be the main wear mechanism of flank wear with the FBU formation. These provide new insights into the wear mechanism in dry cutting of Inconel 718.

Based on the above results, it can be concluded that we can realize the SPT in the dry cutting of Inconel 718 in the cutting speed range of ~30 m/min, especially at a cutting speed of 10 m/min, at which lower tool wear formation, longer tool life, and better surface roughness can be obtained. A larger feed rate can lead to a higher BUL, which may result in a reduction in surface roughness, but a higher BUL can improve the protective effect of the BUL and extend the tool life. Therefore, it is recommended to use a larger feed rate to maximize the utilization of the BUL under the premise of ensuring surface quality. Since the material microstructure has a major effect on the BUL formation condition, we can also expect the development of a free-machining Inconel 718 by actively and purposely utilizing the BUL.

## Figures and Tables

**Table 1:** Chemical composition of workpiece, Inconel 718(AG) and Inconel 718(ST) (wt. %).

Inconel 718(AG)				Inconel 718(ST)			
Ni	53.07	Nb + Ta	5.28	Ni	52.43	Nb + Ta	5.30
C	0.03	Ti	0.94	C	0.03	Ti	0.98
Mn	0.04	Al	0.58	Mn	0.06	Al	0.59
Si	0.04	Cu	0.03	Si	0.08	Cu	0.07
Cr	18.57	Fe	18.2	Cr	18.46	Fe	18.49
Co	0.09	Other	Bal	Co	0.31	Other	Bal
Mo	3.05			Mo	2.9		

**Table 2:** Physical and mechanical properties of Inconel 718(AG) and Inconel 718(ST).

Material	Inconel 718(AG)	Inconel 718(ST)
Density ρ [$kg/m^{3}$]	8200	
Melting point $T_{m}$ [℃]	1300	
Thermal conductivity k [W/(m·K)]	11	
Specific heat c [J/(kg·K)]	440	
Tensile strength $\sigma_{T}$ at 25 °C [MPa]	1455 ^(a)^	924 ^(b)^
Elongation L at 25 °C [%]	21 ^(a)^	55 ^(b)^
Tensile strength $\sigma_{T}$ at 650 °C [MPa]	1160 ^(a)^	×
Elongation L at 650 °C [%]	33.5 ^(a)^	×
Vickers hardness HV [HV(0.1/10)]	530 ± 18 ^(c)^	358 ± 13 ^(c)^

^(a)^ Data from material certification; ^(b)^ Data from ref. [40]; ^(c)^ Measured experimental data.

**Table 3:** Cutting conditions.

Cutting Conditions	Values
Cutting-edge radius *R* [μm]	2.5, 80, 165
Cutting speed V [m/min]	10, 30, 50, 70, 90
Feed rate f [mm/rev]	0.001, 0.002, 0.005, 0.01, 0.02, 0.05, 0.1, 0.2
Cutting depth w [mm]	0.5, 1.0, 2.0
Cutting time t [s]	1, 10, 30, 60, 120, 180, 240, 300
Cutting atmosphere	Dry (Without cool air or cutting fluid supply); Air (Cool air is supplied to cutting point); Wet (Cutting fluid is supplied to cutting point and workpiece)

## Data Availability

All relevant data can be obtained in this article.
